# Ultrafast Charge Transfer Dynamics of Thioflavin T Probed by Time-Resolved Raman Spectroscopy

**DOI:** 10.1021/acsphyschemau.6c00006

**Published:** 2026-04-07

**Authors:** Sebok Lee, Taehyung Jang, Jongwon Im, Yoonsoo Pang

**Affiliations:** Department of Chemistry, 65419 Gwangju Institute of Science and Technology, 123 Cheomdangwagi-ro, Buk-gu, Gwangju 61005, Republic of Korea

**Keywords:** intramolecular charge transfer, thioflavin T, stimulated Raman spectroscopy, excited-state dynamics, structural dynamics

## Abstract

Thioflavin T (ThT) is a commonly used dye that probes amyloid fibrils associated with Alzheimer’s and Parkinson’s diseases. Binding to amyloid fibrils significantly enhances ThT fluorescence, while hindering the formation of the twisted intramolecular charge transfer (ICT) state in the dimethylaniline group. On the other hand, ThT fluorescence is strongly quenched in aqueous and aliphatic alcohol solutions with efficient ICT in the excited states. No direct experimental evidence for the structural changes of ThT with ICT has been reported yet. In this work, we report the structural changes of ThT during ICT by time-resolved Raman spectroscopic methods, which include the bend of the benzothiazole and the twist of the dimethylaniline group. Both femtosecond stimulated Raman spectroscopy and impulsive stimulated Raman spectroscopy suggest that the ICT coordinate of ThT can be strongly coupled with the low-frequency deformation modes. Time-dependent density functional theory calculations and the evaluated vibrational reorganization energies between the ground and excited states strongly support our experimental results based on time-resolved vibrational spectroscopy.

## Graphical abstract

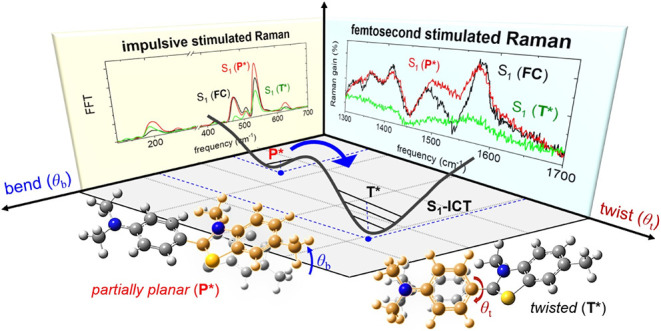

## Introduction

Thioflavin T (ThT) is well-known as a fluorescence probe for amyloid fibrils, often found in patients with Alzheimer’s and Parkinson’s.^1–5^ Fluorescence of ThT is predominantly quenched in polar solvents with the intramolecular charge transfer (ICT) process, whereas its binding to amyloid fibrils inhibits ICT and results in strong fluorescence.^6–10^ Furthermore, the ICT dynamics of ThT are highly sensitive to confined environments, such as reverse micelles or elevated solution viscosity, suggesting that ThT may act as a molecular rotor.^6,11–14^ Numerous theoretical and experimental works have suggested that the ICT dynamics in polar solvents are associated with a twist (θ_t_) of the dimethylaniline group, resulting in decreased fluorescence.^6–13,15–19^ Although femtosecond transient absorption (TA) and fluorescence spectroscopy have been widely employed to investigate the excited-state dynamics of ThT, direct experimental evidence for structural changes accompanying ICT remains limited.^6–10^ Recently, Joo and coworkers proposed an intermediate state possibly existing in the ICT of ThT based on the coherent oscillations obtained from femtosecond fluorescence upconversion measurements, where the bend angle (θ_b_) of the benzothiazole ring off the plane of the dimethylaniline group increases from zero (the planar structure in the S_1_ locally excited (LE) state).^7^ Twist (θ_t_) of the dimethylaniline group along the central C–C bond is suggested to follow the initial bend of the benzothiazole ring to complete the structural changes of the ICT. Time-dependent density functional theory (TDDFT) calculations at the CAM-B3LYP/6-31G­(d,p) level predicted the structure of twisted ICT state (T*; θ_t_ = 90° and θ_b_ = 27°; see Figure 1a). In addition, a stable partially planar geometry (P*; θ_t_ = 14° and θ_b_ = 17°) has been suggested by the TDDFT calculations at the CAM-B3LYP/6-31G­(d,p) and other levels.^7,16^ Mukherjee et al. proposed a dual ICT bifurcation model involving the twisting of both the dimethylaniline and dimethylamino groups, based on observations of ThT in methanol and chloroform solvents with similar viscosity but different polarity.^15^ They suggested that the strong fluorescence observed in chloroform may arise from the rotation of the dimethylamino group based on TDDFT calculation.^15^ However, most theoretical studies have focused on the evaluation of absorption and emission spectra consistent with experimental results, while TDDFT simulations of excited-state geometries remain challenging and often yield inconsistent justifications due to strong dependence on the choice of functional, basis set, and solvent model.

**Figure 1:**
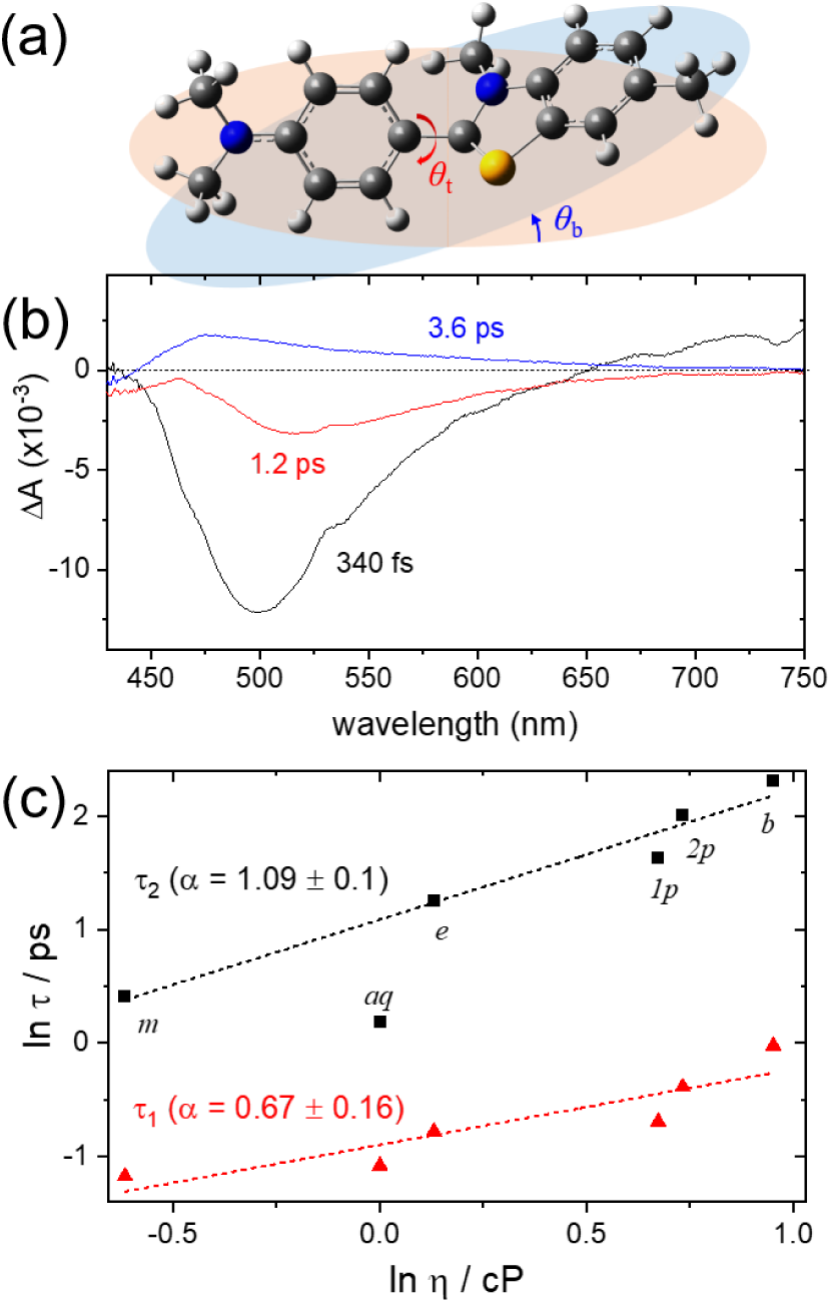
(a) Molecular structure of thioflavin T (ThT), where θ_t_ denotes the twist angle of dimethylaniline group and θ_b_ the bend angle of benzothiazole group, (b) evolution-associated difference spectra (EADS) of ThT in water obtained from the global analysis of femtosecond transient absorption (TA) results with the 403 nm excitation, (c) solvent viscosity dependence of two time constants τ_1_ and τ_2_ of ThT obtained from TA measurements (*m*: methanol, *aq*: water, *e*: ethanol, *1p*: 1-propanol, *2p*: 2-propanol, *b*: 1-butanol).

Time-resolved Raman spectroscopy has been widely used to investigate the ultrafast structural dynamics of chromophores during various photophysical processes in excited states.^20–30^ The ICT dynamics of *push–pull* chromophores with the backbones of stilbene, biphenyl, styrylpyran, and styrylpyridinium have been investigated by femtosecond stimulated Raman spectroscopy (FSRS), where the time-resolved vibrational probe with both high spectral (<10 cm^–1^) and temporal resolutions (<50 fs) retrieved the chromophores’ structural changes, such as the twist of electron-donating or accepting moieties.^23,26,28,29^ The broadband Raman probe, which completes the stimulated Raman process combined with the narrowband Raman pump, provides a wide spectral range often covering most of the fingerprint frequency region.^31–33^ However, the remaining femtosecond fundamental pulse in the Raman probe, generated in white-light continuum generation of the fundamental pulse, often limits the efficient spectral measurements in a low-frequency region (<800 cm^–1^) of Stokes Raman spectrum.^33,34^ On the other hand, impulsive stimulated Raman spectroscopy (ISRS) has been introduced as an alternative time-domain Raman spectroscopy, where the oscillatory components of the Raman-pump-induced absorption changes are obtained using the identical Raman probe. The frequency-domain Raman spectrum, including the low-frequency region, is obtained by the Fourier transformation of the temporal modulations in the Raman probe.^25,35–39^ In this work, the multimodal structural dynamics of ThT, including the bend of the benzothiazole ring and the twist of the dimethylaniline group are explored by the time-resolved vibrational methods of FSRS and ISRS.

## Experimental Section

### General

ThT (TCI Chemicals, Tokyo, Japan) and all solvents were used without further purification. The steady-state absorption spectra were measured in a UV/vis absorption spectrometer (Mega-900, Scinco, Seoul, Korea), and the steady-state emission spectra were measured in a home-built time-correlated single photon counting (TCSPC) based on a TCSPC module (Picoharp 300, PicoQuant, Berlin, Germany) with 405 nm excitation (P-C-405, PicoQuant).

### Transient Absorption and Femtosecond Stimulated Raman Setup

The details of transient absorption^40,41^ and femtosecond stimulated Raman setups^28,29^ based on a 1 kHz Ti:sapphire regenerative amplifier (805 nm, <50 fs) were described elsewhere. The actinic pump pulses at 403 nm (∼80 nJ/pulses) were generated by second harmonic generation (SHG) in a 0.1 mm thick β-barium borate (BBO) crystal and compressed by a pair of chirped mirrors (−30 ± 10 fs^2^ group delay dispersion; Layertec GmbH, Mellingen, Germany). The probe pulses generated by white-light generation in a 3 mm thick sapphire window (Eksma Optics, Vilnius, Lithuania) were measured in a fiber-based CCD spectrometer (QE65Pro, Ocean Optics, Largo, FL, USA) for transient absorption measurements.

The narrowband picosecond Raman pump pulses at 802 nm (∼500 nJ/pulses, <10 cm^–1^) were generated by a grating filter (1200 gr/mm) and the broadband Raman probe pulses (830–930 nm) generated by supercontinuum generation in a 4 mm-thick YAG crystal were measured by a multichannel CCD detector (PIXIS 100, Princeton Instruments, Trenton, NJ, USA) attached to an f = 320 mm spectrograph (Triax 320, Horiba Jobin Yvon, Tokyo, Japan). All dilute 60 μM solutions in a 2 mm-thick quartz cuvette were measured for transient absorption spectra. A 1 mM solution of ThT in water and 1-propanol in a 1 mm-thick quartz flow cell was used for stimulated Raman measurements. The samples were recirculated by a peristaltic pump to minimize the photodamage.

### Time-Resolved Impulsive Stimulated Raman Setup

The experimental setup of time-resolved impulsive stimulated Raman (Figure 2) is based on a 1 kHz Ti:sapphire regenerative amplifier. The actinic pump pulses at 403 nm (∼500 nJ/pulses) are generated by SHG in a 0.2 mm-thick BBO crystal. Raman pump and probe pulses were generated in a home-built noncollinear optically parametric amplifier (NOPA). In the NOPA, the pump pulses (403 nm) generated by the SHG in a BBO crystal (θ = 29.2°, ϕ = 90°, 0.2 mm thick, Eksma) and the seed pulses generated by a sapphire window (2 mm thick, Eksma) were overlapped at the second BBO crystal (θ = 27.5°, ϕ = 90°, 1 mm thick, Eksma). The output pulses from the NOPA in the 490–560 nm range (see inset spectrum in Figure 2a) were compressed to ∼20 fs pulsewidth by a chirped mirror pair (Layertec GmbH, Mellingen, Germany) with a fine-tuning of the dispersion by a wedge prism pair (23RQ12-02-M, Newport). After splitting two pulses by a 1 mm-thick beamsplitter (Layertec GmbH, Mellingen, Germany) with a low GDD dispersion, Raman pump and probe pulses were sent to the sample by adding some delay using a reflector mounted on the piezo stage (P-629.1CD, Physik Instrumente), moving with an interval of 10 fs time. The Raman pump pulses were modulated by an optical chopper (MC2000, Thorlabs, Newton, NJ, USA). The Raman probe pulses, split into two channels of signal and reference and dispersed by a 1200 gr/mm grating, were detected by two photodiodes (S2281-01, Hamamatsu) connected to the low-noise current preamplifiers (SR570, Stanford Research Systems, Sunnyvale, CA, USA). The small changes in the Raman probe pulses were improved by introducing the reference channel and boxcar averager (SR250, Stanford Research Systems). To minimize the group delay dispersion of pulses, the thin fused-silica flow cell with a thickness of 500 μm (48/UTWA2, Starna Cells) was adopted for ISRS measurements. A 2 mM solution of ThT in solvents was used for ISRS measurements. No sample aggregations were observed in 1–2 mM concentrations used for FSRS and ISRS measurements.

**Figure 2:**
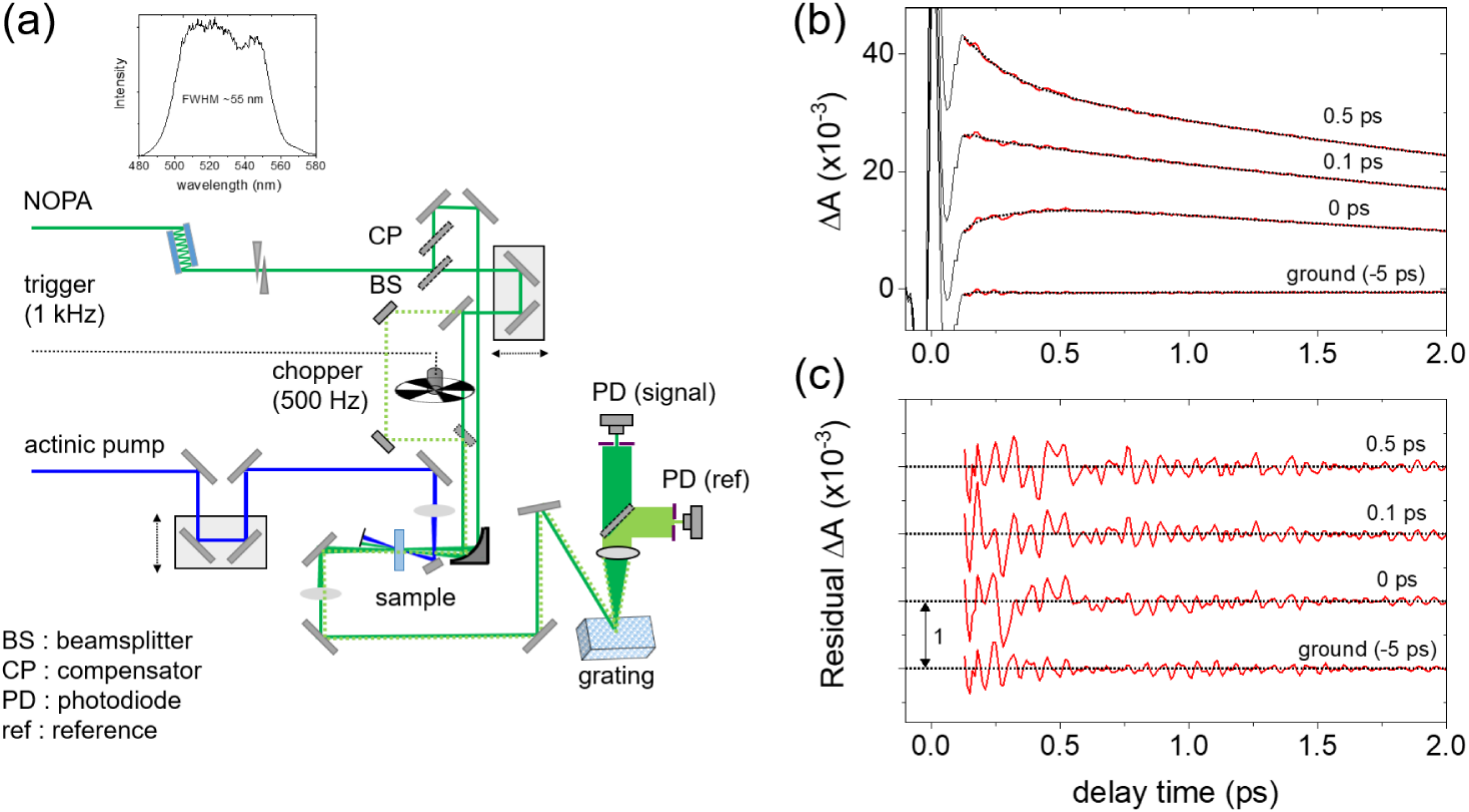
(a) Impulsive stimulated Raman setup with the Raman pump/probe spectrum (inset), (b) temporal responses of the Raman probe obtained from the interferometric measurements with the identical Raman pump, at various time delays between the actinic pump and the Raman pump, (c) residual temporal response data obtained by subtracting exponential decay functions and removing early time delay (<0.1 ps) data contaminated with nonlinear coherent artifacts.

## Results and Discussion

### Femtosecond Transient Absorption Measurements

ThT dissolved in water and aliphatic alcohols exhibits strong ICT characters in the S_1_ excited state, with large Stokes’ shifts of 3340–3460 cm^–1^ (absorption and emission spectra are available in the Supporting Information; Figure S1). The TA spectra of ThT in aqueous and aliphatic alcohol solutions were obtained with 403 nm excitation. Although the TA spectra appear similar among all the solvents, the ICT dynamics and the excited-state lifetimes of the S_1_-ICT state show strong solvent dependence. The global analysis with the sequential model retrieved three kinetic components called evolution-associated difference spectra (EADS) from the TA results. The TA spectra and EADS results of ThT are available in Figures S2–S3 in the Supporting Information, and Table 1 summarizes the excited-state dynamics of ThT from TA measurements.

**Table 1:** Excited-State Kinetics of ThT from the TA Results

Solvent	τ_1_ (ps)	τ_2_ (ps)	τ_3_ (ps)	Viscosity (mPa·s; 20 °C)
Water	0.34	1.2	3.6	1.00
Methanol	0.31	1.5	21	0.54
Ethanol	0.46	3.5	22	1.14
1-Propanol	0.50	5.1	65	1.96
2-Propanol	0.68	7.4	66	2.08
1-Butanol	0.98	10.0	360	2.59

Figure 1b shows three EADS obtained from TA spectra of ThT in aqueous solution. The fastest (340 fs) EADS represents the locally excited (LE) state or the Franck–Condon (FC) region, where the positive band in the 650–750 nm is the excited-state absorption (ESA) and the strong negative band centered at 500 nm represents the stimulated emission (SE). The intermediate (1.2 ps) EADS with the substantially decreased SE band red-shifted to 515 nm, represents the intermediate S_1_-LE′ state. The slowest (3.6 ps) EADS shows a new ESA band at 475 nm without the SE bands representing the S_1_-ICT state. The excited-state dynamics of ThT have similarly been observed in water and several polar solvents, although the time resolutions of TA or fluorescence upconversion measurements used in these works vary.^6,7,9,17^ As shown in Figure 1c, the time constants τ_1_ and τ_2_, representing the relaxation and ICT dynamics in the S_1_ state, respectively, both show strong solvent-viscosity dependence among water and aliphatic alcohols. The slope α shows the viscosity dependence of the excited-state kinetics following the Kramer–Smoluchowski relationship (eq 1),

1 $$\tau_{i}=Z_{i}\eta^{-\alpha_{i}}\exp\left(-E_{a}/k_{B}T\right)$$

where τ_i_ represents the excited-state lifetime, η the solvent viscosity, Z_i_ the pre-exponential factor, and E_a_ the activation barrier of the transition.^42–45^ We obtained a smaller α_1_ = 0.67 for the vibrational relaxation in the S_1_-LE state and larger α_2_ = 1.09 for the ICT dynamics. It is known that barrier-less transitions often show small slopes (α < 0.7) in the Kramer–Smoluchowski plot.^19,44,46^ Thus, TA results represent that ThT undergoes the barrier-less relaxations (τ_1_) and ICT (τ_2_) along the multidimensional nuclear coordinates, including the twist of dimethylaniline and the bend of benzothiazole group, in the S_1_ state. Solvent viscosity-dependent ICT dynamics of ThT have been extensively reported.^6,7,12,13,15,16,18,19^ Ultrafast solvation dynamics (0.17 ps in aqueous solution and 0.59 and 9.5 ps in 1-propanol) were observed from the dynamic Stokes shifts of the SE bands (Figure S4 in the Supporting Information), where the fastest components are relevant to the vibrational relaxation dynamics in the S_1_-LE state. However, our TA results provide the first evidence that the vibrational relaxation dynamics (τ_1_) leading to the intermediate S_1_-LE′ state are also strongly dependent on solvent viscosity, thereby validating the structural evolution of ThT from the S_1_-LE or FC state.

### Femtosecond Stimulated-Raman Spectroscopy Measurements

The structural changes of ThT upon the ICT have been further explored by time-resolved vibrational measurements. To reveal the structural evolution during the ICT process, including the formation of the S_1_ intermediate state, accompanied by the bending of the benzothiazole moiety and subsequent twisting of the dimethylaniline group, 1-propanol was primarily used as the solvent. Figure 3a shows the FSRS results of ThT in 1-propanol obtained with 403 nm excitation, where each excited-state Raman spectrum was subtracted from the ground-state Raman spectrum obtained at −5 ps delay and the transient backgrounds were removed by fitting a low-order polynomial function. The excited-state Raman spectra of ThT differ from the ground-state spectrum with the appearance of several vibrational bands in the 1300–1650 cm^–1^ range. The appearance of strong excited-state Raman intensities over the weak ground-state signals indicates that the excited-state Raman signals are strongly enhanced by the resonance Raman effects of the Raman pump (at 802 nm) in resonance with the S_1_-LE ESA band in the 650–750 nm (Figure 1b). Thus, no ground-state bleaching is observed in FSRS results (Figure 3a).

**Figure 3:**
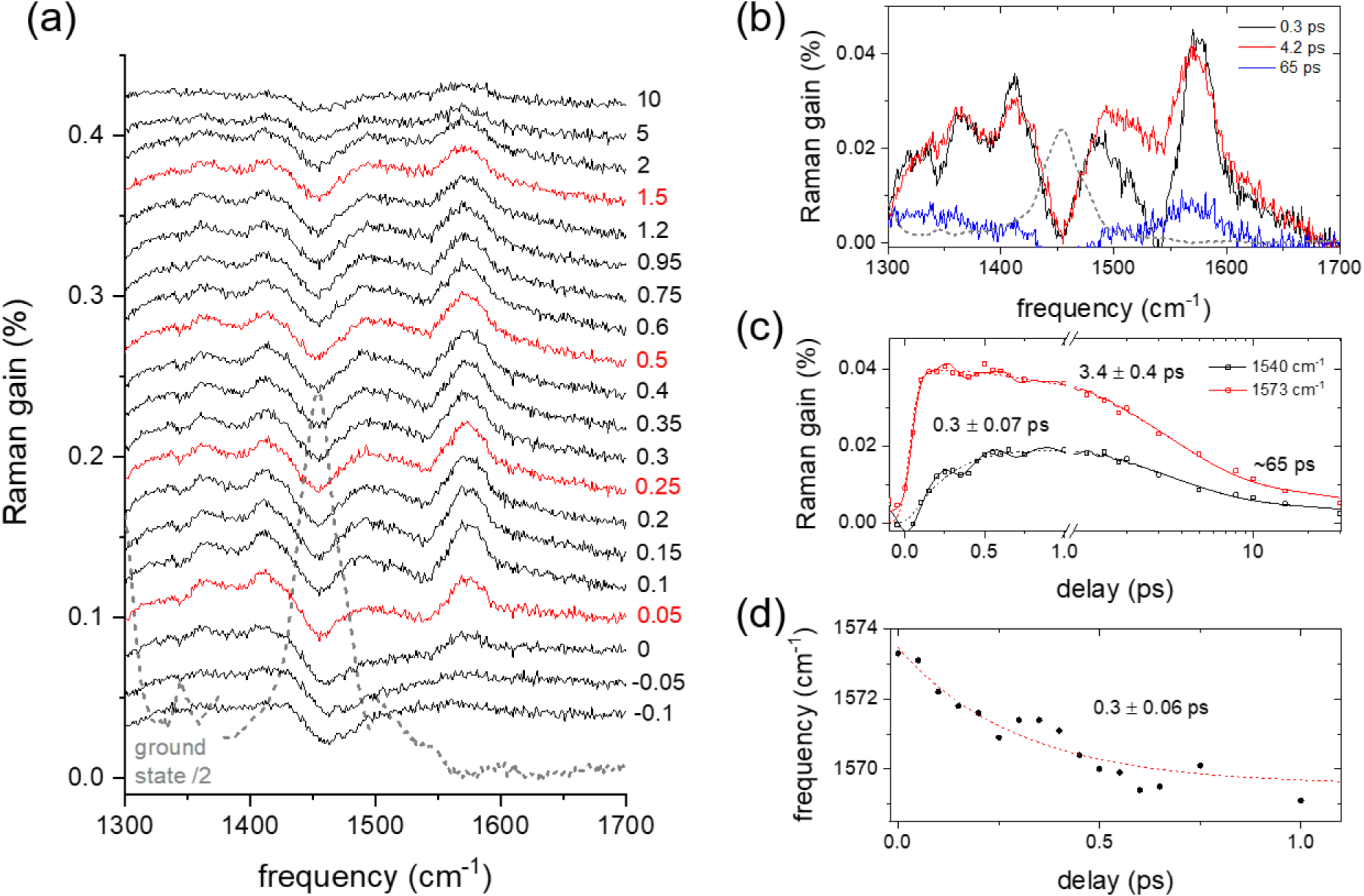
FSRS results of ThT in 1-propanol obtained with 403 nm excitation; (a) time-resolved spectra, (b) EADS obtained from the global analysis with a sequential model, (c) population dynamics, and (d) frequency shifts of the major excited-state Raman bands. The dashed lines in panels (a) and (b) represent the ground-state spectrum. Solid and dashed lines in panels (c) and (d) represent the kinetic fits obtained with and without the coherent oscillation signals, respectively.

The global analysis of the FSRS results was performed with the sequential model by Glotaran program.^47^ The EADS shown in Figure 3b exhibits several excited-state Raman bands which spectrally overlap in the 1475–1600 cm^–1^ range. Three kinetic components (0.3, 4.2, and 65 ps) were resolved from the global fit analysis based on the sequential model. The fastest (0.3 ps) kinetic component of ThT represents the relaxation dynamics of the S_1_-LE state, and the subsequent 4.2 and 65 ps components are the ICT dynamics and population decay of the ICT state, respectively, consistent with the TA results (Figure S3d in the Supporting Information). The EADS with the time constants of 0.3 and 4.2 ps show a clear difference in the 1480–1570 cm^–1^ range with the apparent increase around 1540 cm^–1^. The population dynamics compared in Figure 3c clearly show the ∼0.3 ps increase and decrease in the Raman intensities of 1540 and 1573 cm^–1^ bands, respectively, along with the coherent oscillations with periods of ∼0.34 and ∼0.17 ps. The vibrational frequencies of 99 and 196 cm^–1^ were retrieved by Fourier transforming the coherent oscillations in the 1540 and 1573 cm^–1^ bands, respectively (see Figure S5 in the Supporting Information). These low-frequency oscillations observed in FSRS may indicate the vibrational wavepackets created by the actinic pump among the vibronic bands of the S_1_-LE state.^48–50^ It is also possible that the ICT coordinate of ThT is considered strongly coupled with the low-frequency structural deformation modes, specifically the out-of-plane bending motions localized on the benzothiazole moiety.^21,29,49,51–53^ Further experimental or theoretical investigations are essential to unambiguously determine the origin of these coherent oscillation signals in the skeletal vibrational bands of ThT.

Vibrational assignments of the ground and excited-state Raman spectra of ThT were based on (TD)­DFT calculations. The excited-state Raman band at 1573 cm^–1^ is assigned as the ν_8a_ (ph, bt[Fn fn1]) while the broad overlapping band centered at 1540 cm^–1^ is considered as the ν_8b_ (bt) modes based on the TDDFT calculations (Figure S16 in the Supporting Information). The ν_8a_ mode involves a symmetric in-plane stretching of the aromatic ring, resembling a breathing-like motion, while the ν_8b_ mode corresponds to an asymmetric in-plane deformation with alternating C–C/C=C bond stretching. The peak positions of the 1573 cm^–1^ band shown in Figure 3d exhibit compatible (∼0.3 ps) red shifts with the population kinetics. The blue shifts and band narrowing of the excited-state vibrational modes are generally expected during vibrational cooling associated with the loss of excitation energy to the anharmonic bath.^54,55^ Thus, these spectral changes in the skeletal vibrational modes of ThT with the ultrafast (∼0.3 ps) dynamics would be interpreted as the structural evolutions of ThT in the S_1_ state, including the bend of benzothiazole and the twist of the dimethylaniline group. This will be discussed further in later sections.

### Impulsive Stimulated Raman Measurements

To better understand the multidimensional ICT coordinate of ThT, including the low-frequency deformations strongly coupled to the skeletal vibrational modes of benzothiazole and dimethylaniline, we further investigated the ICT dynamics of ThT by ISRS measurements. The temporal responses of the Raman probe pulses were measured interferometrically using identical Raman pump and probe. The time-domain interferometric signals were extracted by subtracting a sum of exponential functions, and the signals with minimal nonlinear coherent artifacts (after 0.1 ps delay; see Figure 2b–c) were used in the spectral conversion by a fast Fourier transformation (FFT). The Hanning window function and zero padding were used to obtain the frequency-domain vibrational spectra, called FFT power spectra.^35,56^ The excited-state difference spectra were obtained by subtracting the ground-state (at −5 ps) spectrum from the excited-state spectra obtained at each time delay. The intensity and center frequency changes of major Raman bands were fit with the sum of the exponential-Gaussian convoluted functions and exponential functions, respectively, to retrieve the excited-state dynamics of ThT.

Figure 4 shows the ISRS results of ThT in 1-propanol obtained with 403 nm excitation. Since the Raman pump pulses in the ISRS measurements are resonant with both the SE band of the S_1_-LE state and the ESA band of the S_1_-ICT state, the excited-state Raman intensities are enhanced and ground state bleaching bands were not observed. The ground-state spectrum of ThT in 1-propanol (Figure 4a) includes the in-plane deformations at 474, 505, and 539 cm^–1^, while the δ_C–C/C–O_ band of solvent 1-propanol appears at 467 cm^–1^. The vibrational assignments are based on the DFT calculations (Figure S15 in the Supporting Information). The excited-state spectra of ThT in 1-propanol (Figure 4a) with 200, 473, 505, 537, and 624 cm^–1^ bands differ from the ground-state spectrum and exhibit distinct growth and decay dynamics among these modes. The global analysis for the intensities of these modes (Figure 4b) retrieves two fast time constants of 0.37 and 3.4 ps in addition to the lifetime of the ICT state (65 ps from TA results). The ultrafast relaxation (0.37 ps) and the subsequent ICT dynamics (3.4 ps) in the S_1_ state are compatible with the TA and FSRS results. Interestingly, the intensities of excited-state Raman bands, including the ν_oop_ (bt) + $\delta_{CH_{3}}$ at 200 cm^–1^, ν_ip_ (bt) + $\delta_{\mathrm{CH/C}H_{3}}$ at 537 cm^–1^, and ν_ip_ (ph) + ν_oop_ (bt) at 624 cm^–1^, increase with the 0.37 ps time constant, while the ν_ip_ (ph) + $\delta_{CH_{3}}$ at 473 cm^–1^ and ν_ip_ (bt) + $\delta_{\mathrm{CH/C}H_{3}}$ at 505 cm^–1^ show the corresponding decreases. The former Raman bands (at 200, 537, and 624 cm^–1^) represent ThT in the intermediate ICT state, while the latter (at 473 and 505 cm^–1^) are considered as the FC bands. It is noted that the 200 and 537 cm^–1^ bands were commonly observed from the oscillating components in the TA and time-resolved fluorescence results.^7,10^ The vibrational frequencies of the major bands (at 473, 505, and 537 cm^–1^) show ultrafast (0.51 and 4.6 ps) shifts (Figure 4c), similar to the population dynamics (0.37 and 3.4 ps) of the excited-state bands. The red shifts (0.51 ps) observed in these bands correspond to the structural evolution of ThT toward the intermediate S_1_-LE′ state, whereas the structural changes during ICT, such as the twist of the dimethylaniline group, lead to slight blue shifts (4.6 ps) in these bands. The population and frequency changes of these Raman bands suggest that the ICT of ThT in the excited state would occur in two steps, including the bend of benzothiazole (θ_b_) and the twist of dimethylaniline group (θ_t_).^7,16^ The time-resolved Raman measurements (FSRS and ISRS) reveal significant vibrational details, offering insights into the structural changes of ThT upon the ICT in the excited states.

**Figure 4:**
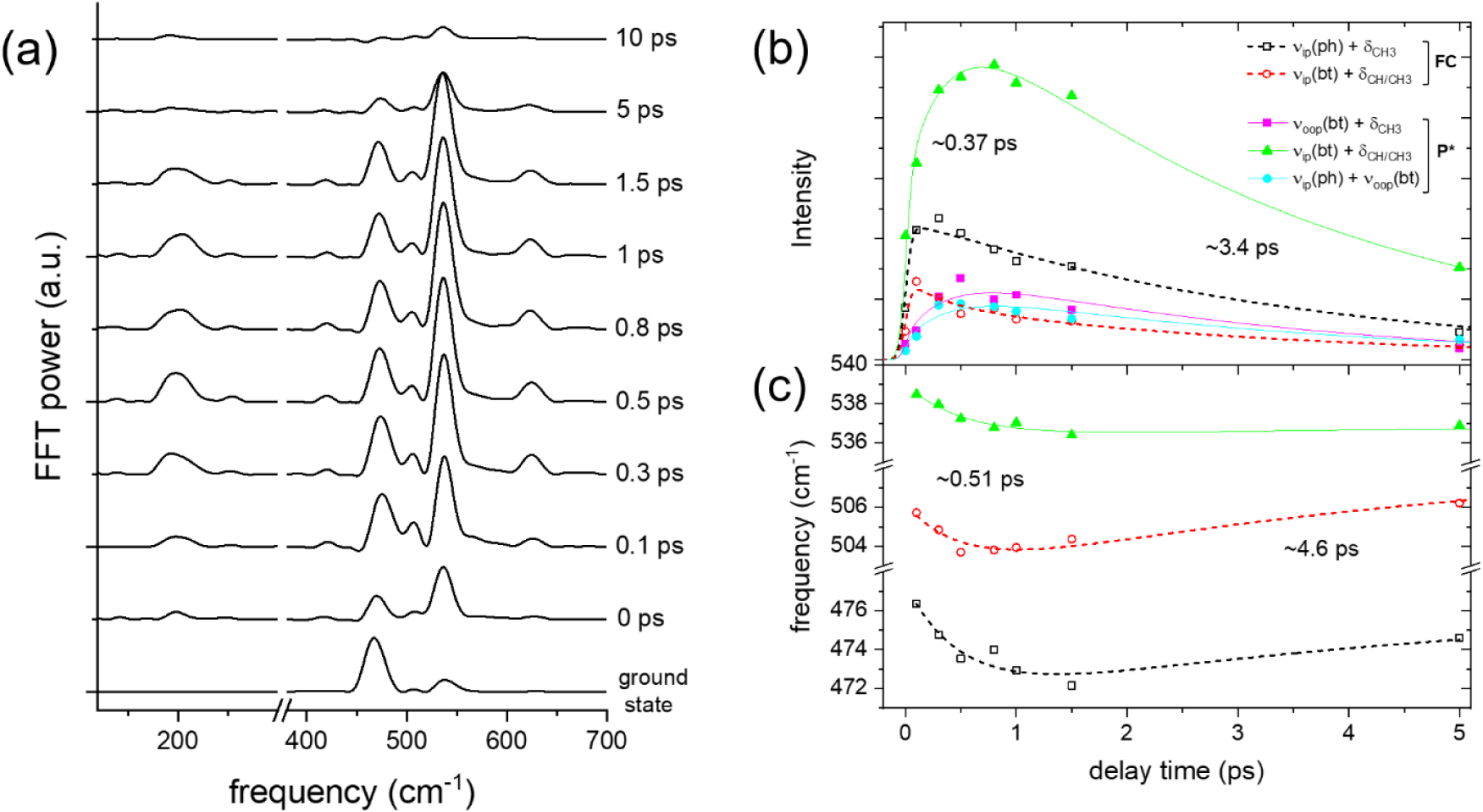
ISRS results of ThT in 1-propanol obtained with 403 nm excitation; (a) time-resolved spectra processed via FFT power transformation, (b) population dynamics, and (c) frequency shifts of the major excited-state Raman modes. Filled symbols with solid lines in panels (b) and (c) represent the intermediate S_1_-LE′ modes, and hollow symbols with dashed lines denote the Franck–Condon (**FC**) modes. Global fit results for intensity and frequency changes are displayed as solid or dashed lines.

The structural changes of ThT in other aliphatic alcohols and water were similarly observed by ISRS (Figures S6–S9 in the Supporting Information). Much faster (∼100 fs and 1.2 ps in methanol) or similar (380 fs and 2.9 ps in 2-propanol) dynamics, compared to the 1-propanol case, were obtained from aliphatic alcohol solutions. However, the formation of the intermediate (S_1_-LE′) state was not resolved in aqueous solution due to a fast time constant, where the excited-state decay (∼500 fs) of the 537 cm^–1^ band was only resolved. The solvent-dependent kinetics of the ν_ip_ (bt) + $\delta_{\mathrm{CH/C}H_{3}}$ mode at 537 cm^–1^ in the intermediate (S_1_-LE′) state were compared in Figure S9.

### Structural Changes of ThT during ICT

We performed TDDFT calculations to obtain the molecular structures and Raman spectra of ThT in the excited states. Two optimized geometries in the S_1_ state, including the P* geometry (θ_t_ = 19.4° and θ_b_ = 16.2°) and T* geometry (θ_t_ = 91.6° and θ_b_ = 27.1°), were obtained depending on the level of TDDFT calculations, including the CAM-B3LYP/6-311G­(d,p) and B3LYP/6-311G­(d,p) by using the conductor-like polarized continuum model (CPCM) for 1-propanol. The vibrational reorganization energy, vibrational frequencies, and Raman intensities were calculated from the DFT calculations at the same level as used in the geometry optimizations.^57–59^ The details of the (TD)­DFT calculations are available in the Supporting Information.

Figure 5 compares the ISRS spectra of ThT in 1-propanol obtained at 0.1, 0.5, and 10 ps with the calculated vibrational reorganization energies of ThT in the partially planar (P*) and the twisted ICT (T*) geometries, which are obtained from projections onto the ground-state geometry (Figure S11 in the Supporting Information compared the optimized geometries of ThT in the ground and excited states). Vibrational reorganization energies, obtained from the Huang–Rhys factors, are known as a sensitive measure of structural displacements between two states and account for the contribution of each vibrational mode.^57–59^ Even though resonance Raman theory describes Raman intensities based on Franck–Condon factors between the initial and final states, vibrational reorganization energies can provide complementary insights by quantifying the contribution of each vibrational mode to structural relaxation. Therefore, comparing experimental ISRS spectra with calculated reorganization energies offers a meaningful approach to interpreting structural dynamics, particularly when the direct calculation of resonance Raman intensities is not feasible. The intermediate (S_1_-LE′) spectrum obtained at 0.5 ps closely resembles the calculated vibrational spectrum of the partially planar (P*) structure, where the ISRS bands at 200, 537, and 624 cm^–1^ show substantial growth from the FC spectrum at 0.1 ps, as summarized in Table S4 in the Supporting Information. This suggests that the bending of benzothiazole may be the first structural change associated with the ICT of ThT in the S_1_ state, assigning the partially planar (P*) structure to ThT in the intermediate (S_1_-LE′) state. However, the relaxed ICT spectrum obtained at 10 ps appears dissimilar to the calculated vibrational spectrum for the T* structure. ThT fluorescence is quenched in polar solvents and twisted geometries associated with structural changes during ICT in the excited state have been proposed, as reported in many references.^8,11,13,15,18,19^ The frontier molecular orbital diagrams of ThT in the ground state, as well as in the P* and T* geometries in the S_1_ state (Figure S12), indicate that the T* geometry leads to fluorescence quenching due to its negligible oscillator strength (∼0.003). Although the relaxed ICT spectrum observed at 10 ps appears inconsistent with the calculated vibrational spectrum of the T* structure, the experimentally observed fluorescence quenching suggests that the relaxed S_1_-ICT state may involve a contribution from the T* geometry.

**Figure 5:**
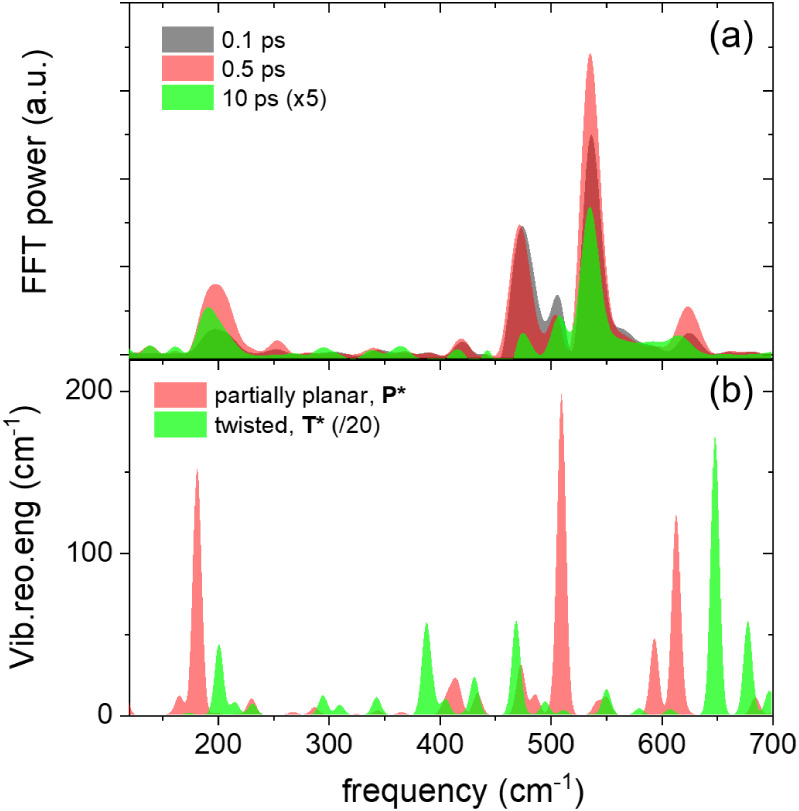
(a) ISRS spectra of ThT in 1-propanol at 0.1, 0.5, and 10 ps, (b) vibrational reorganization energies of ThT in the partially planar (P*) and the twisted ICT (T*) geometries in the S_1_ state evaluated from the ground state geometry. The optimized geometry of P* was obtained from the (TD)­DFT calculation at the CAM-B3LYP/6-311G­(d,p) level with the CPCM (1-propanol), and the B3LYP exchange-correlation functional was used instead for the T* and ground state geometry optimizations.

The spectral evidence for the P* and T* geometries of ThT in the S_1_ state can also be obtained from FSRS results (Figure 3b), where the second (4.2 ps) and the third (65 ps) EADS may represent the P* and T* geometries, respectively. Compared to the first (0.3 ps) EADS associated with the FC geometry, the P* spectrum exhibits increased spectral contributions around 1540 cm^–1^, while clear band separation remains unfeasible due to strong neighbor bands at 1490 and 1570 cm^–1^. The spectral contributions around 1540 cm^–1^ are considered as the ν_8b_ (bt) according to the TDDFT calculations for the P* geometry (Figure S16 in the Supporting Information). However, the spectral identity for the T* geometry of ThT in the S_1_ state remains unclear. TDDFT calculations estimate the spectral separation of ν_8a_ (ph) and ν_8a_ (bt) bands with the twisting of the dimethylaniline group, but the third EADS (65 ps) from FSRS measurements exhibits a single band at 1570 cm^–1^. Nevertheless, considering the pronounced fluorescence quenching of ThT in polar solvents, the involvement of the T* geometry, arising from the twisting of the dimethylaniline group, remains a plausible interpretation of the relaxed S_1_-ICT state, even if its vibrational signature is not clearly resolved in the FSRS measurements.

## Conclusions

Combined time-resolved Raman spectroscopic methods, FSRS and ISRS, revealed spectral evidence for the structural changes of ThT occurring with the ICT in the S_1_ state. Previous TA and fluorescence upconversion results suggested two-step ICT dynamics, including the bending of the benzothiazole group and the twisting of the dimethylaniline group. From the combined experimental results of FSRS and ISRS of ThT, structural evolution dynamics of 300 ∼ 370 fs and 3 ∼ 4 ps have been separated along with the corresponding Raman bands. The intermediate structure of ThT in the S_1_ state formed with the fast (300–370 fs) dynamics shows the in-plane and out-of-plane deformation bands at 200, 537, and 624 cm^–1^ and the skeletal vibrational modes at ∼1540 cm^–1^, being compatible with the previous TA and fluorescence upconversion results. TDDFT calculations with various exchange-correlation functionals and basis sets have confirmed that the intermediate structure would represent the partially planar geometry (P*; θ_b_ = 16.2° and θ_t_ = 19.4°) with the bend of the benzothiazole group. However, the vibrational signatures of the subsequent twisted geometry (T*; θ_b_ = 27.1° and θ_t_ = 91.6°) with the twist of the dimethylaniline group estimated from TDDFT calculations were not clearly resolved in either FSRS or ISRS measurements. Our experimental findings provide a valuable framework for extending investigations into the structural dynamics of ThT bound to amyloid fibrils, where its fluorescence is significantly enhanced, and would contribute to a deeper understanding of how molecular conformation governs fluorescence quantum yield.

## Supplementary Material



## Footnotes

1 “ph” denotes phenyl and “bt” represents benzothiazole.

## Notes

Absorption and emission; TA; FSRS; and ISRS results of ThT; (TD)­DFT simulations for the excited-state geometries and Raman spectra (PDF)

## ◆

National Center for Quantum Technology Strategy, Korea Research Institute of Standards and Science, 267 Gajeong-ro, Yuseong-gu, Daejeon 34113, Republic of Korea
