# Supplementary material for: Ultrafast Charge Transfer Dynamics of Thioflavin T Probed by Time-Resolved Raman Spectroscopy
Source: ACS Phys Chem Au. 2026 Apr 7;6(4):678–86. doi: 10.1021/acsphyschemau.6c00006 (PMC13397442; doi:10.1021/acsphyschemau.6c00006)
Supplement: Supplementary file 1 [file pg6c00006_si_001.pdf]

[Supporting Information]

Ultrafast Charge Transfer Dynamics of Thioflavin T Probed by Time-Resolved Raman Spectroscopy

Sebok Lee, Taehyung Jang,[†] *Jongwon Im, and Yoonsoo Pang**

Department of Chemistry, Gwangju Institute of Science and Technology, 123 Cheomdangwagi-ro, Buk-gu, Gwangju 61005, Republic of Korea

*E-mail: ypang@gist.ac.kr

[†]National Center for Quantum Technology Strategy, Korea Research Institute of Standards and Science, 267 Gajeong-ro, Yuseong-gu, Daejeon 34113, Republic of Korea

Table of Contents

1. Steady-state absorption and emission spectra of ThT S2
2. Femtosecond transient absorption (TA) results S3
3. Femtosecond stimulated Raman spectroscopy (FSRS) results S8
4. Impulsive stimulated Raman spectroscopy (ISRS) results S9
5. (TD)DFT simulations for the excited state geometries and Raman spectra S15
6. References S28

1. Steady-state absorption and emission spectra of ThT

The steady-state absorption and emission spectra of ThT dissolved in water and aliphatic alcohols are shown in Figure S1. The absorption band of ThT centered at 410 nm in aqueous solution shows polarity-dependent red-shifts (416 → 419 nm) in aliphatic alcohols (methanol → 1-butanol). The emission bands of ThT in all solvents show large Stokes' shifts of 3340-3460 cm^{-1}, indicating the strong ICT character of the S_1 excited state.

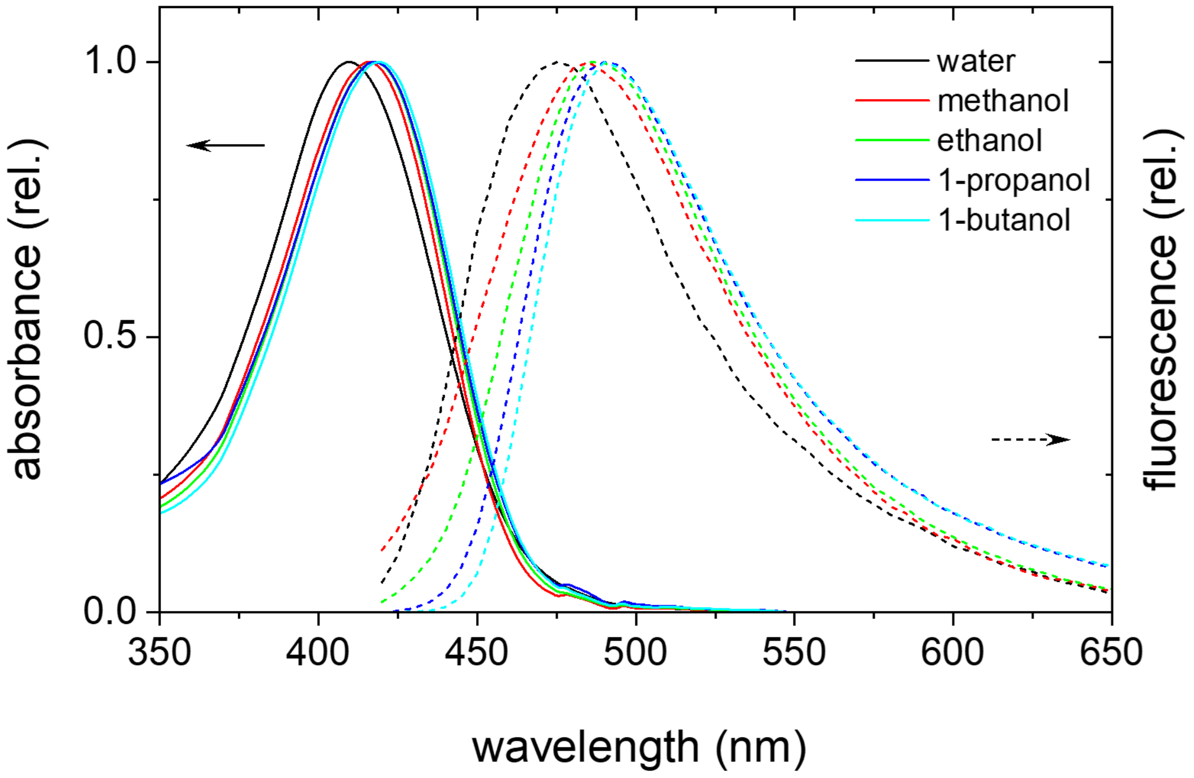

Figure S1. Steady-state absorption (solid lines) and emission (dotted lines) spectra of ThT in water and aliphatic alcohols. The 405 nm excitation was used for emission measurements.

2. Femtosecond transient absorption (TA) results

Transient absorption results (surface plots and difference spectra at some time delays) of ThT dissolved in water and aliphatic alcohols are shown in Figure S2. In water, the excited-state absorption (ESA) bands in the 650−750 nm and the stimulated emission (SE) bands centered at around 490 nm appear at early time delays (<0.1 ps), which is assigned as the locally-excited (LE) state or Franck-Condon region. As the time delay increases, the new ESA bands of the S_1-ICT state at around 462 nm replace the ESA bands at 650−750 nm with further blue-shifts to 450 nm. The SE bands at 490 nm in early time delays red-shift to 515 nm with the decrease of intensity, which is considered as the ICT and the population decay to the ground state of the S_1-ICT state. The spectral changes in transient absorption results of aliphatic alcohols were quite similar to the water results, while the ICT dynamics of ThT are strongly dependent on solvent viscosity. The transient absorption results of ThT in water and aliphatic alcohols were analyzed by the global analysis with a sequential model using a software package Glotaran.[1] Figure S3 shows the evolution-associated difference spectra (EADS) of ThT in water and aliphatic alcohols obtained from the global analysis. The excited-state dynamics of ThT in aqueous solution are explained by three kinetic components of τ_1 = 340 fs, τ_2 = 1.2 ps, and τ_3 = 3.6 ps: 340 fs EADS represents the S_1-LE or Franck-Condon state, 1.2 ps EADS represents the intermediate (S_1-LE′) state, and 3.6 ps EADS is the relaxed S_1-ICT state. The excited-state dynamics of ThT in aliphatic alcohols were similarly summarized by three kinetic components, representing the relaxation and ICT dynamics in the S_1 state, and population decay of the S_1-ICT state, respectively. Two kinetic components, τ_1 and τ_2, exhibit strong dependence on solvent viscosity. This indicates that both processes involve structural evolutions of ThT, including the bending of the benzothiazole and twisting of the dimethylaniline group, as suggested in the literature.[2-6]

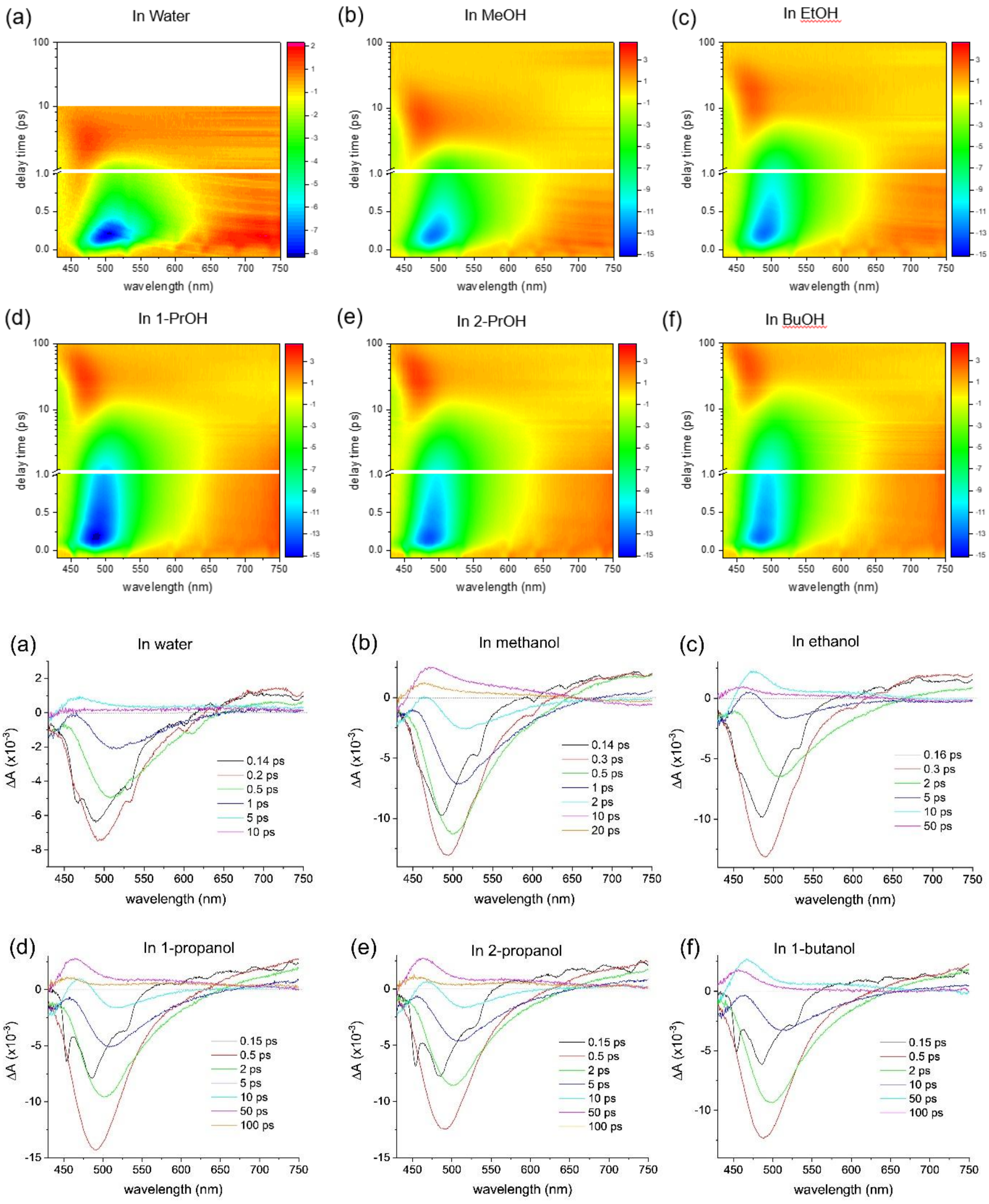

Figure S2. Transient absorption results (surface plots and difference spectra at some time delays) of ThT in (a) water, (b) methanol, (c) ethanol, (d) 1-propanol, (e) 2-propanol, and (f) 1-butanol with 403 nm excitation.

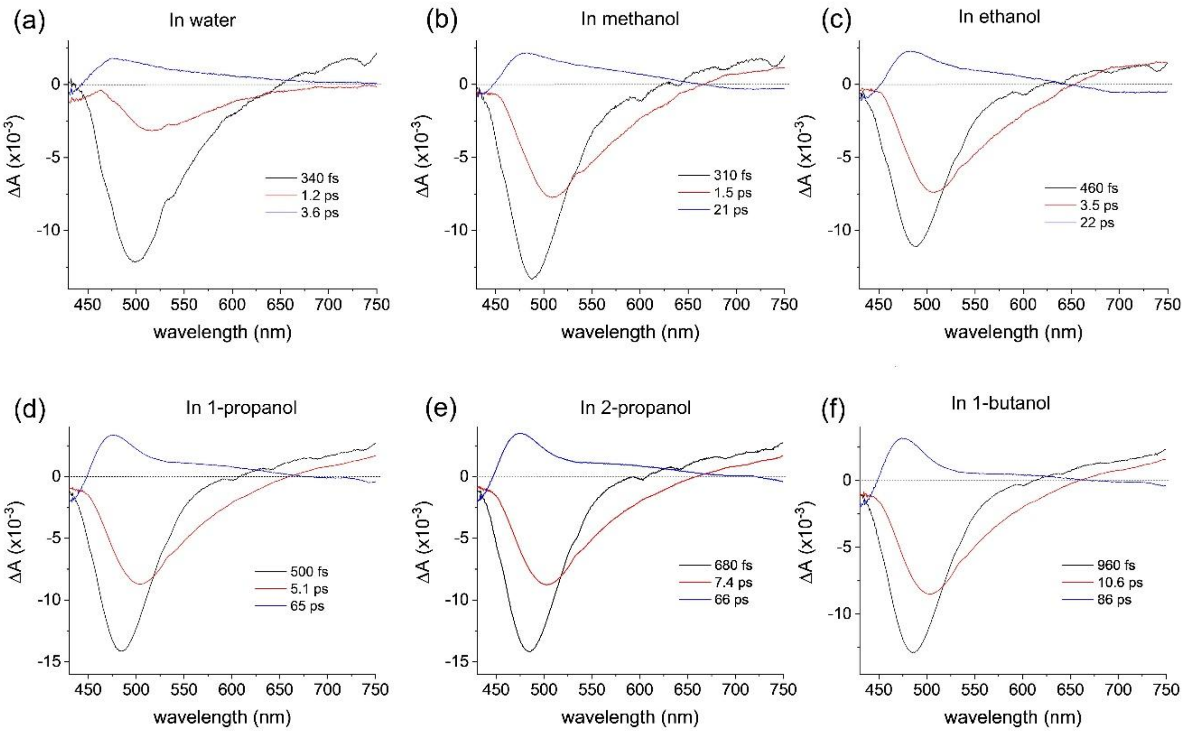

Figure S3. The evolution-associated difference spectra (EADS) obtained from the global analysis of the transient absorption results of ThT in (a) water, (b) methanol, (c) ethanol, (d) 1-propanol, (e) 2-propanol, and (f) 1-butanol with 403 nm excitation.

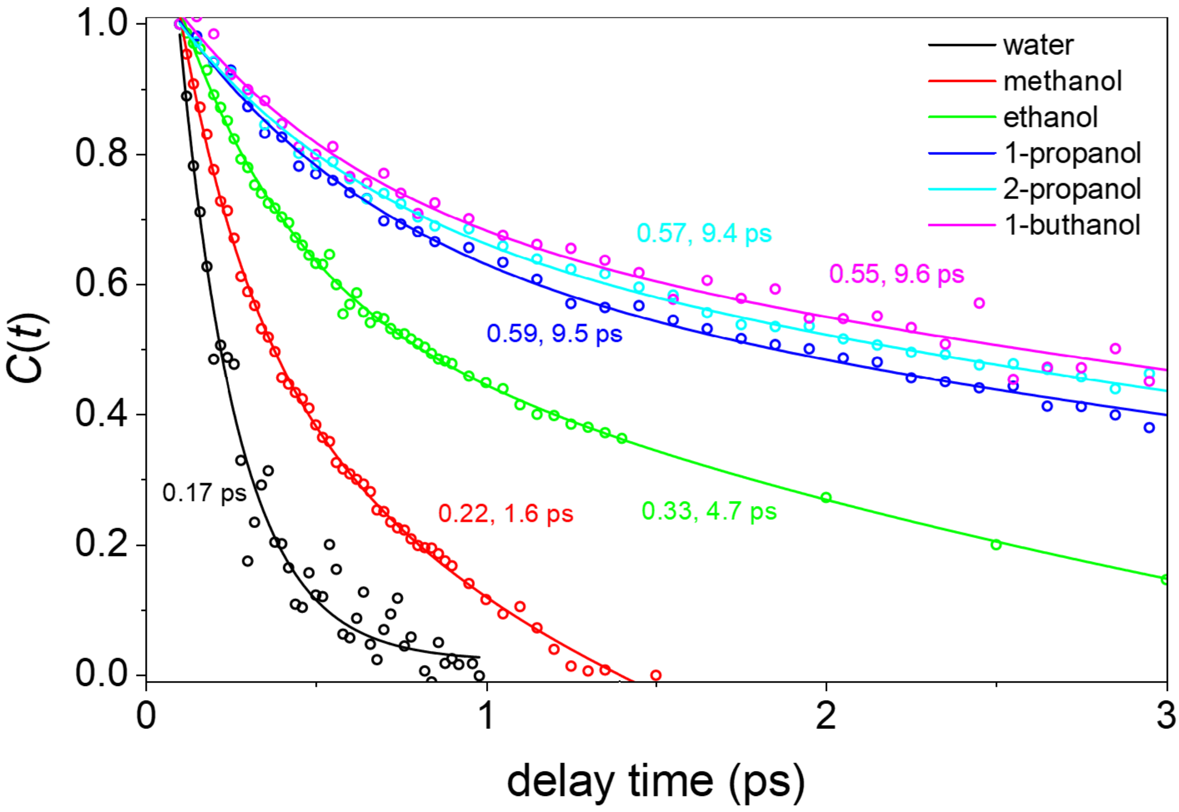

Figure S4. The dynamic Stokes shifts of ThT in the SE bands are obtained as the frequency-frequency correlation function (FFCF) $C(t)=\left(\nu(t)-\nu(\infty)\right)/\left(\nu(0)-\nu(\infty)\right)$, where $\nu(t)$ represents the maximum emission frequency at time delay t. Table S1 summarizes the kinetic analysis.

Table S1. Dynamic Stokes shifts of ThT from the TA results

solvent	a_1	τ_1 (ps)	a_2	τ_2 (ps)	$<\tau>$ (ps)[a]
water	1.00	0.17	-	-	0.17
methanol	0.32	0.22	0.68	1.6	1.2
ethanol	0.30	0.33	0.70	4.7	3.4
1-propanol	0.27	0.59	0.73	9.5	7.1
2-propanol	0.21	0.57	0.79	9.4	7.6
1-butanol	0.21	0.55	0.79	9.6	7.6

[a] Amplitude average lifetime, $\langle \tau \rangle = \frac{a_1\tau_1 + a_2\tau_2}{a_1 + a_2}$

3. Femtosecond stimulated Raman spectroscopy (FSRS) results

The coherent oscillation signals in the FSRS of ThT were fit to the sum of the exponential-Gaussian convoluted functions and several sinusoidal functions multiplied with an exponential damping function (eq. S1),

$$\begin{aligned}\Delta_{\text{Raman Gain}}(t) = A_0 + \sum_i A_i \exp\left(\frac{\omega^2}{2\tau_i^2} - \frac{t-t_0}{\tau_i}\right)\left[1 - erf\left(\frac{\omega^2 - \tau_i(t-t_0)}{\sqrt{2}\omega\tau_i}\right)\right] \\ + \sum_i B_i \exp\left(\frac{\omega^2}{2\tau_{D,i}^2} - \frac{t-t_{osc,0}}{\tau_{D,i}}\right)\left[1 - erf\left(\frac{\omega^2 - \tau_{D,i}(t-t_{osc,0})}{\sqrt{2}\omega\tau_{D,i}}\right)\right]\sin\left(2\pi\frac{t-t_{osc,0}}{\tau_{osc,i}}\right)\end{aligned} \quad \text{(S1)}$$

where $t_{osc,0}$ is the position of time zero for the damped oscillation functions, $\tau_{D,i}$ and $\tau_{osc,i}$ are the time constant for damping and the period of each oscillation component, respectively.

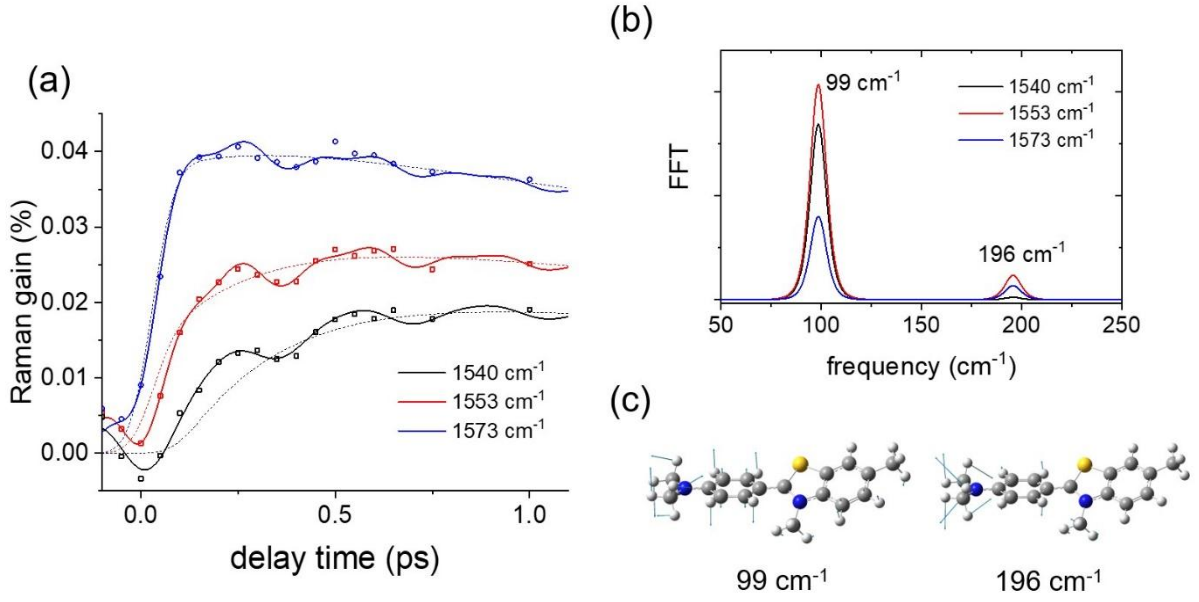

Figure S5. (a) Coherent oscillation signals in the major vibrational modes of ThT in 1-propanol from FSRS results, (b) fast Fourier transformation (FFT) results of the coherent oscillation signals, and (c) low-frequency out-of-plane deformation modes of ThT from the optimized geometry in the S_1 excited state with the partially planar geometry.

4. Impulsive stimulated Raman spectroscopy (ISRS) results

The excited-state dynamics of ThT in 2-propanol (Figure S6) are very similar to the 1-propanol results as the solvent properties of 2-propanol, including solvent viscosity, are quite similar to those of 1-propanol. The vibrational assignments of ThT in 2-propanol were based on the DFT and TDDFT simulations performed in the ground and the S_1 excited states. The ground-state spectrum of ThT in 2-propanol includes the in-plane deformations (505 and 537 cm^{-1}), in addition to the solvent mode, the $\delta_{C\text{–}C/C\text{–}O}$ (485 cm^{-1}). The excited-state spectra of ThT in 2-propanol show the similar ICT dynamics (0.38 and 2.9 ps from global analysis of all the excited-state bands) as the 1-propanol results. The intermediate (S_1-LE′) bands, ν_{oop} (bt) + δ_{CH3} (203 cm^{-1}), ν_{ip} (bt) + $\delta_{CH/CH3}$ (537 cm^{-1}), and ν_{ip} (ph) + ν_{oop} (bt) (623 cm^{-1}) show the intensity rises with the 0.38 ps time constant, while the Franck-Condon (**FC**) modes, ν_{ip} (ph) + δ_{CH3} (486 cm^{-1}) and ν_{ip} (bt) + $\delta_{CH/CH3}$ (507 cm^{-1}) show the corresponding decreases. In addition, the major bands in the 470–540 cm^{-1} range show ultrafast (0.54 and 6.6 ps) peak shifts, similar to the two kinetic components (0.38 and 2.9 ps) from the population dynamics.

Figure S7 shows the ISRS of ThT in methanol, which is less viscous than 1-propanol and 2-propanol. The ground-state spectrum of ThT in methanol includes the in-plane deformations (478, 500, and 536 cm^{-1}). The excited-state spectra of ThT in methanol show much faster ICT dynamics (0.1 and 1.2 ps from global analysis of all the excited-state bands) compared to the 1-propanol and 2-propanol results. The **P*** modes, ν_{oop} (bt) + δ_{CH3} (218 cm^{-1}), ν_{ip} (bt) + $\delta_{CH/CH3}$ (538 cm^{-1}), and ν_{ip} (ph) + ν_{oop} (bt) (629 cm^{-1}) increase in intensity with the 0.1 ps time constant, while the **FC** modes, ν_{ip} (ph) + δ_{CH3} (480 cm^{-1}) and ν_{ip} (bt) + $\delta_{CH/CH3}$ (507 cm^{-1}) bands show the corresponding decreases. The peak shifts in the major excited-state Raman bands of ThT in methanol were not observed, may be due to fast (0.1 and 1.2 ps) ICT dynamics.

Figure S8 shows the ISRS of ThT in aqueous solution. The ground-state spectrum shows weak bands around 500–550 cm^{-1}. Upon excitation, the ν_{ip} (bt) + $\delta_{CH/CH3}$ band at 538 cm^{-1} was

only observed with the decay of ~580 fs. The presence of the intermediate (S_1-LE′) state was not determined in aqueous solution, possibly due to much faster ICT dynamics of ThT than the time resolution of the ISRS setup. Lastly, Figure S9 compares the solvent-dependent ICT dynamics of the ν_{ip} (bt) + $\delta_{CH/CH3}$ mode at ~537 cm^{-1} obtained from the ISRS measurements in water, methanol, and 1-propanol.

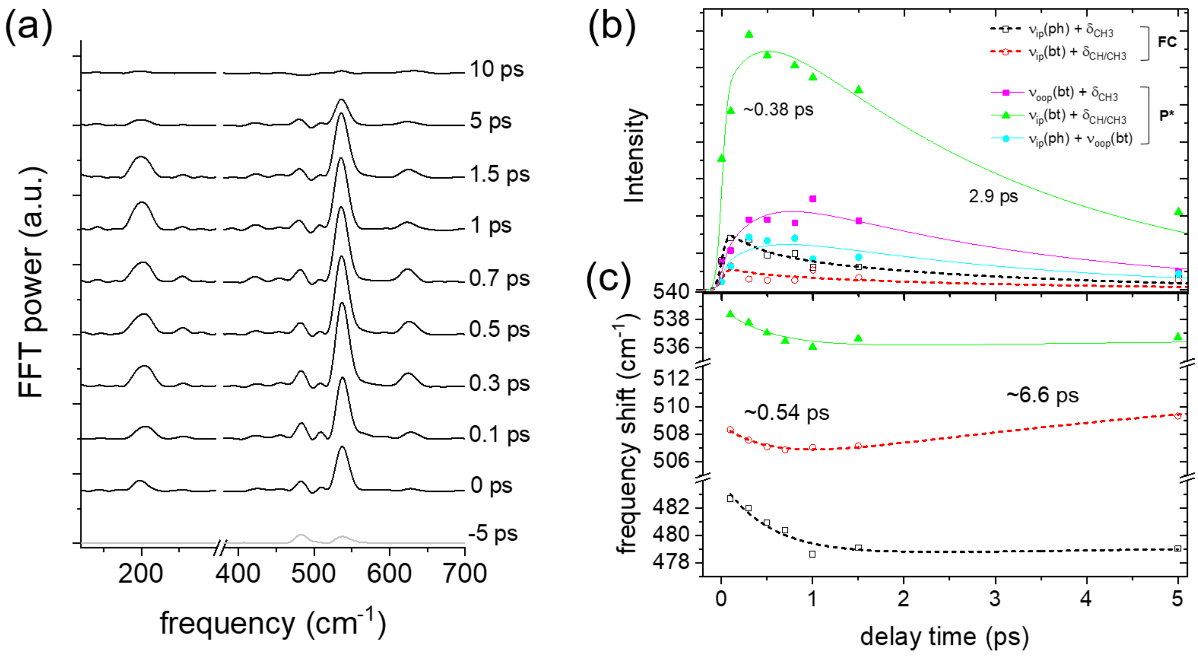

Figure S6. ISRS results of ThT in 2-propanol obtained with 403 nm excitation; (a) time-resolved spectra, (b) population dynamics, (c) frequency shifts of the major excited-state Raman modes. Filled symbols with solid lines in panels (b) and (c) represent the intermediate S_1-LE′ modes, and hollow symbols with dashed lines denote the Franck-Condon (**FC**) modes. Global fit results for intensity and frequency changes are displayed as solid or dashed lines.

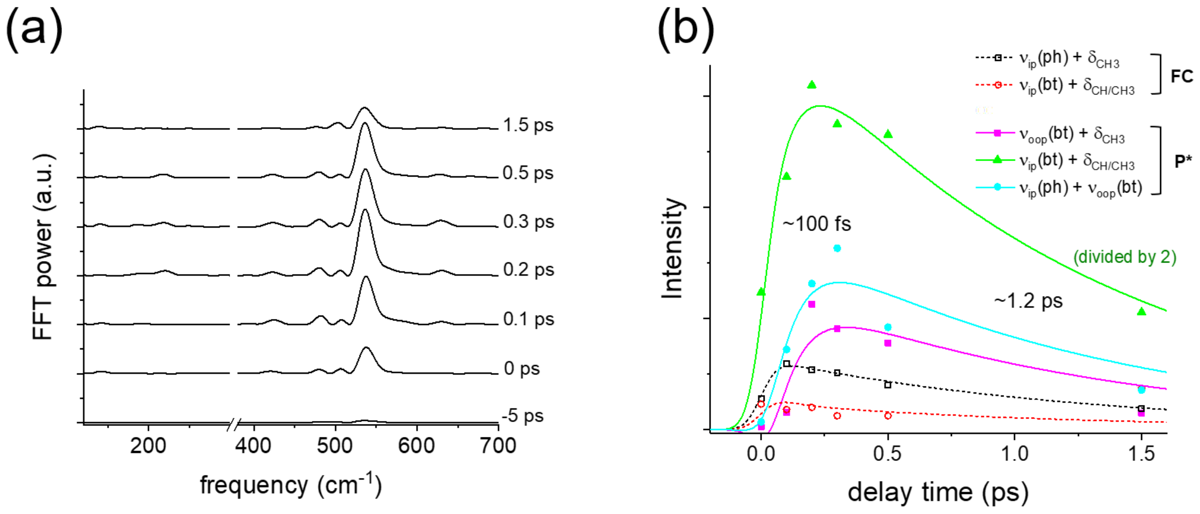

Figure S7. ISRS results of ThT in methanol obtained with 403 nm excitation; (a) time-resolved spectra, (b) population dynamics of the major excited-state Raman modes. Filled symbols with solid lines represent the intermediate (S_1-LE′) modes, and hollow symbols with dashed lines denote the S_1-LE or Franck-Condon (**FC**) modes. Global fit results for intensity are displayed as solid or dashed lines.

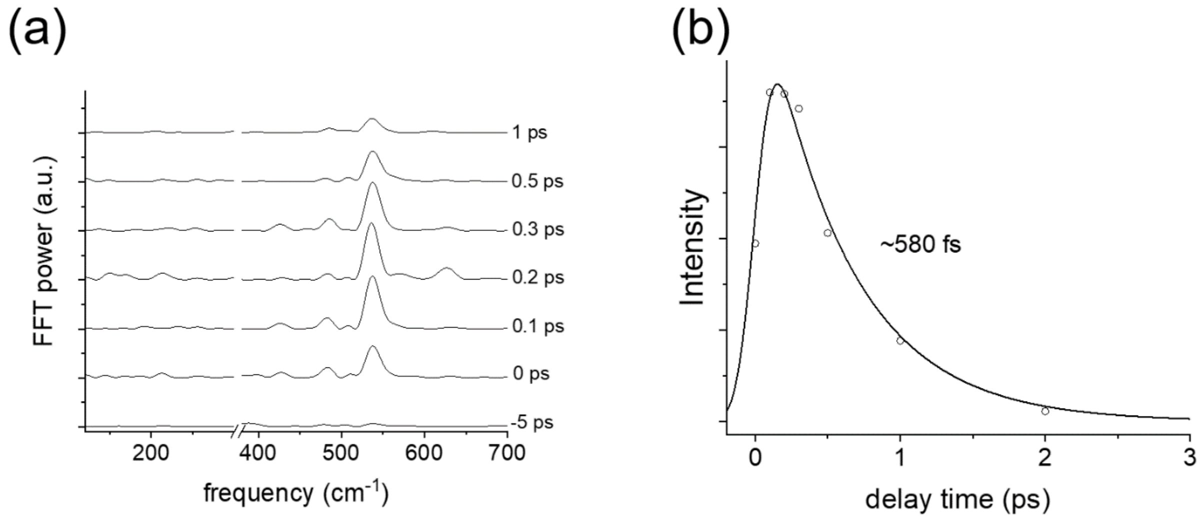

Figure S8. ISRS results of ThT in water obtained with 403 nm excitation; (a) time-resolved spectra, (b) population dynamics of the ν_{ip} (bt) + $\delta_{CH/CH3}$ mode at ~537 cm^{-1} with the Gaussian-exponential convolution fit line.

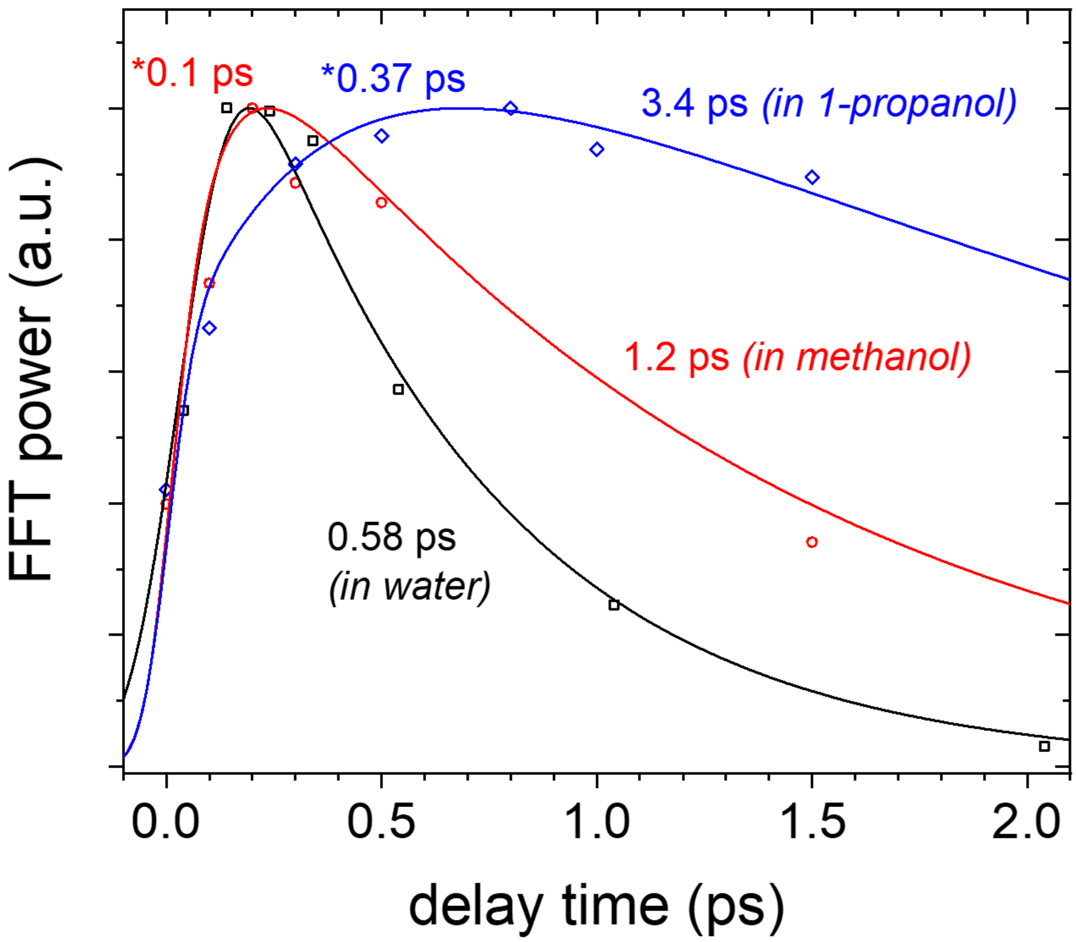

Figure S9. Solvent-dependent ICT dynamics of ThT, retrieved from the population dynamics of the intermediate (S_1-LE′) mode, ν_{ip} (bt) + $\delta_{CH/CH3}$ at ~537 cm^{-1} in the ISRS results, were compared between water, methanol, and 1-propanol solutions.

5. (TD)DFT simulations for the excited state geometries and Raman spectra

Time-dependent density functional theory (TDDFT) simulations were performed by the Gaussian 09 software (Gaussian, Inc., Wallingford, CT, USA).[7] To elucidate the structural changes of ThT during ICT process, including the bend (θ_b) of benzothiazole and the twist (θ_t) of dimethylaniline group (Figure 1a), the pseudo potential energy curves along the rotation of dimethylaniline group (Figure S10) were estimated in the ground (S_0) and the first singlet excited state (S_1). The ground-state geometries of ThT were optimized at the B3LYP/6-311G(d,p) and CAM-B3LYP/6-311G(d,p) levels with the conductor-like polarizable continuum model (CPCM) for solvent effects, resulting in a common ground-state geometry (θ_t =39.5° and θ_b =2.2° in 1-propanol; θ_t =38.8° and θ_b =2.5° in gas phase). The ground-state geometry of ThT obtained from DFT calculations is compatible with the previous results.[3, 5, 6] The pseudo potential energy curves along the rotation of the dimethylaniline group (Figure S10) show little dependence on the DFT level or the CPCM in the ground state. The twisted (θ_t = 90°) dimethylaniline geometry exhibits small energy differences between the gas phase and in 1-propanol solution. On the other hand, the pseudo potential energy curves in the S_1 excited state strongly depend on the exchange correlation functional and solvent effects. With the CPCM (1-propanol), twisted (θ_t = 90°) dimethylaniline geometry is preferred in the S_1 state with the B3LYP functional, while a partially planar (θ_t ~ 20°) structure becomes the S_1 minima with the CAM-B3LYP functional.

Geometry optimization in the S_1 state by the TDDFT calculations strongly depends on the choice of exchange-correlation functional.[3, 5, 6] The twisted dimethylaniline geometry (**T***; θ_t = 90.5° and θ_b = 28.6° in gas phase, and θ_t = 91.6° and θ_b = 27.1° in 1-propanol) is suggested as the S_1 minima at the B3LYP/6-311G(d,p) level. On the other hand, the CAM-B3LYP/6-311G(d,p) level calculations suggest both the twisted (**T***; θ_t = 91.0° and θ_b = 27.8° in gas phase) and partially planar (**P***; θ_t = 19.4° and θ_b = 16.2° in 1-propanol) dimethylaniline

geometries depending on the solvent model. The optimized geometries of ThT in the S_1 state were further calculated using other exchange-correlation functionals, such as M062X,[8] WB97XD,[9] BH&HLYP,[10] and MPWQPW91[11] with the 6-311G(d,p) basis set and CPCM (1-propanol). As summarized in Table S2, the **P*** geometries obtained from the CAM-B3LYP/6-311G(d,p), M062X/6-311G(d,p), WB97XD/6-311G(d,p), BH&HLYP/6-311G(d,p) level calculations are quite similar with the relative energy of 3.07−3.19 eV from the ground state. The **T*** geometries obtained from the B3LYP/6-311G(d,p) and MPWQPW91/6-311G(d,p) levels also appear similar to each other, with the relative energy of 2.47 and 2.69 eV, respectively, from the ground state.

The optimized structures of ThT in the S_1 state are summarized as the **P*** and **T*** geometries obtained by the CAM-B3LYP/6-311G(d,p) and B3LYP/6-311G(d,p) level calculations with the CPCM (1-propanol), respectively, will be used in evaluating the Raman spectra. Figure S11 compares the optimized geometries of ThT in the ground state and the **P*** and **T*** geometries in the S_1 state. The frontier orbital diagrams for the highest occupied molecular orbital (HOMO) and lowest unoccupied molecular orbitals (LUMO) levels of ThT in the ground state, and the **P*** and the **T*** geometry in the S_1 state are shown in Figure S12. The ground-state Raman spectrum of ThT for the optimized geometries of the S_0 state was obtained by the DFT calculations at the B3LYP/6-311G(d,p) level with the solvent CPCM. Figure S13 compares the ground-state experimental and theoretical Raman spectra of ThT. Ordinary Raman spectrum (ORS) obtained with 785 nm excitation, ISRS and FSRS obtained in 1-propanol are compatible with the simulated Raman spectrum (spectral bandwidths of 10 cm^{-1} were used) obtained with CPCM (1-propanol). The FSRS of ThT in 1-propanol appears indistinguishable from the strong solvent vibrational mode at 1455 cm^{-1}. Thus, FSRS spectrum obtained in water was also displayed for comparison. Vibrational frequencies from all the DFT and TDDFT calculations

were rescaled by a factor of 0.967.[12, 13] The vibrational assignments of ThT in the ground state are summarized in Table S3.

The vibrational reorganization energies of ThT in the **P*** (θ_b = 16.2° and θ_t = 19.4°) and **T*** geometries (θ_b =27.1° and θ_t =91.6°) of the S_1 state were calculated by projecting on the reference ground-state optimized geometry in the Dushin program.[14] As shown in Figure S14, the vibrational reorganization energies for the **P*** and **T*** geometries of ThT show little dependence on the TDDFT calculation levels. The vibrational assignments of ThT in the S_1 excited state (**P*** and **T*** geometries) were made based on the frequency shifts and Raman intensity changes from the ground-state spectrum, and summarized in Table S4. Figure S15 shows the major vibrational modes in the low frequency range (120−700 cm^{-1}), while the high-frequency (1500−1700 cm^{-1}) vibrational modes are exhibited in Figure S16.

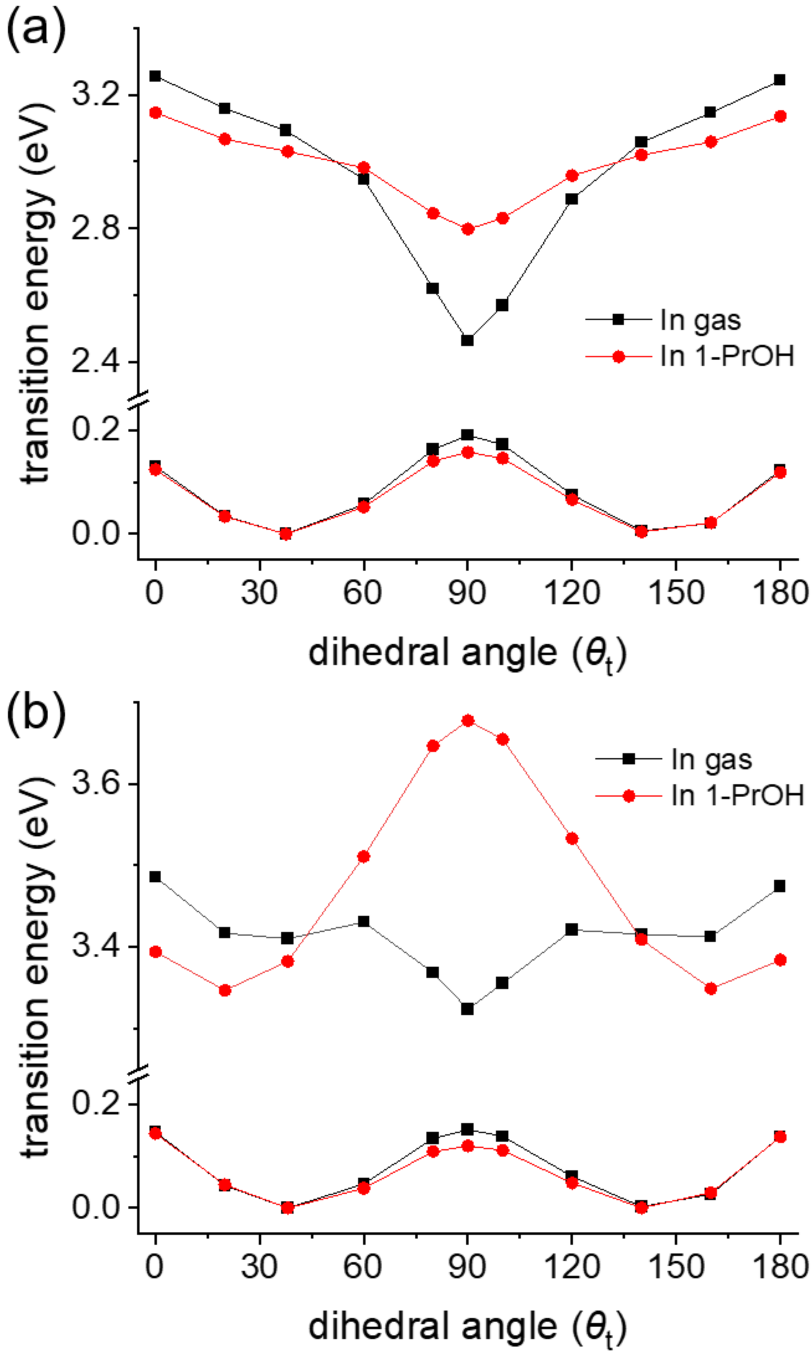

Figure S10. Pseudo-potential energy curve of ThT along the dihedral rotation (θ_t) of the dimethylaniline group. The ground-state optimized geometry and the single point TDDFT calculations at the (a) B3LYP/6-311G(d,p) and (b) CAM-B3LYP/6-311G(d,p) levels with CPCM (1-propanol).

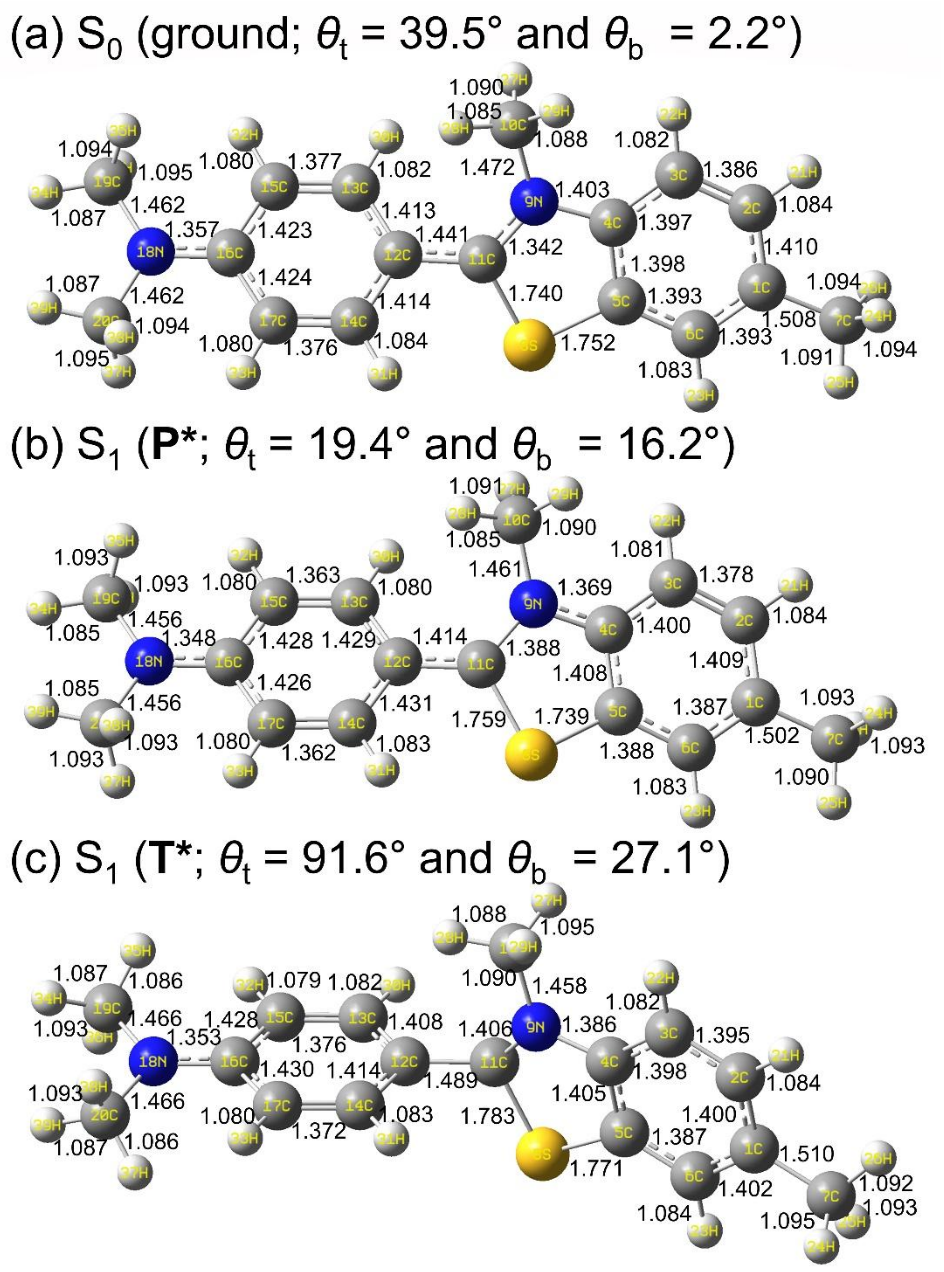

Figure S11. Optimized structures of ThT in (a) the ground state, (b) the partially planar (**P***), and (c) twisted (**T***) geometry in the S_1 state obtained from the DFT/TDDFT calculations with CPCM model (1-propanol). The B3LYP/6-311G(d,p) level was used for the ground state and **T*** geometry, and the CAM/B3LYP/6-311G(d,p) level was used for the **P*** geometry.

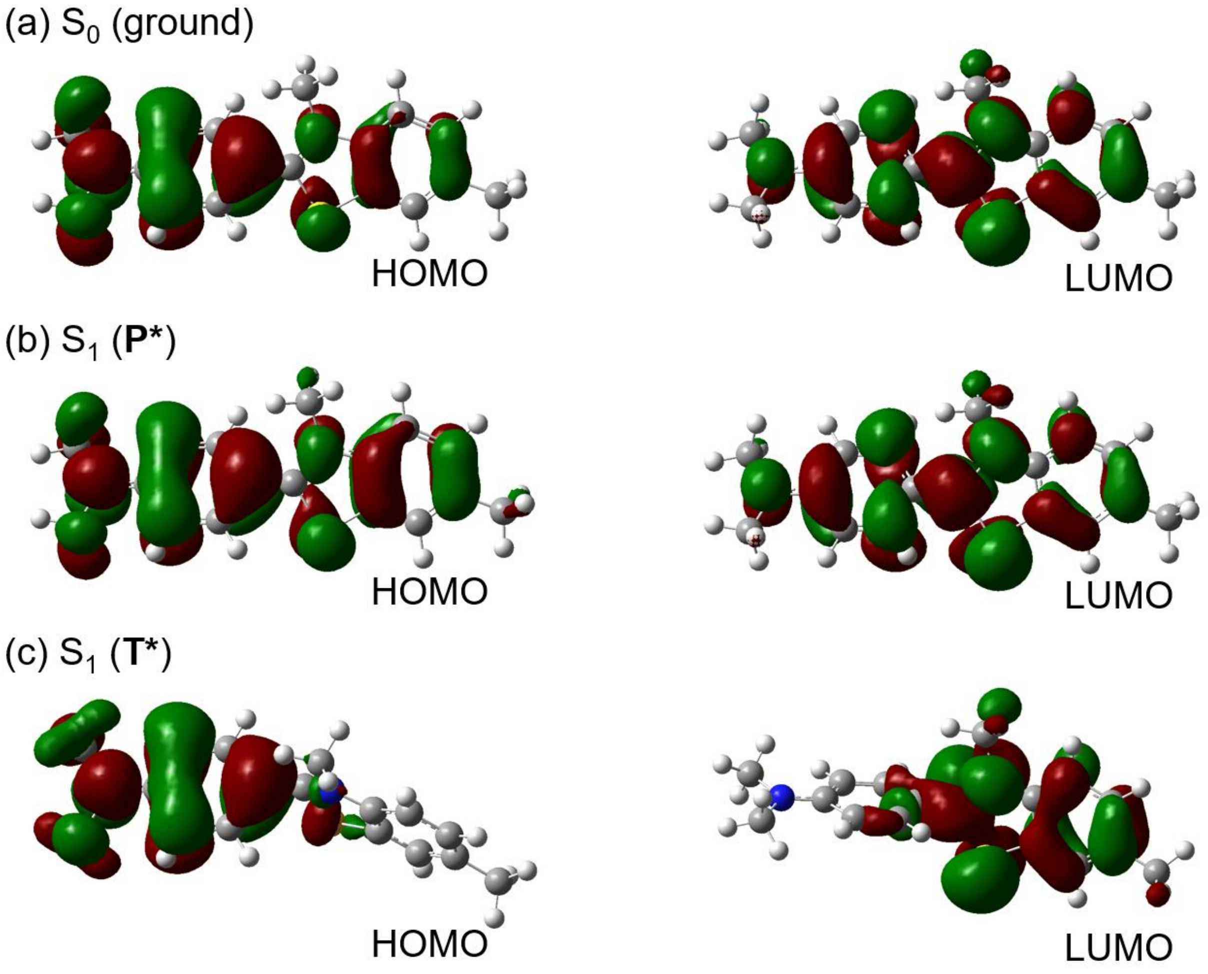

Figure S12. Frontier orbital diagrams for the highest occupied molecular orbital (HOMO) and lowest unoccupied molecular orbitals (LUMO) levels of ThT in (a) the ground state, (b) the partially planar (**P***), and (c) twisted (**T***) geometry in the S_1 state obtained from the DFT/TDDFT calculations with CPCM model (1-propanol). The B3LYP/6-311G(d,p) level was used for the ground state and **T*** geometry, and the CAM/B3LYP/6-311G(d,p) level was used for the **P*** geometry.

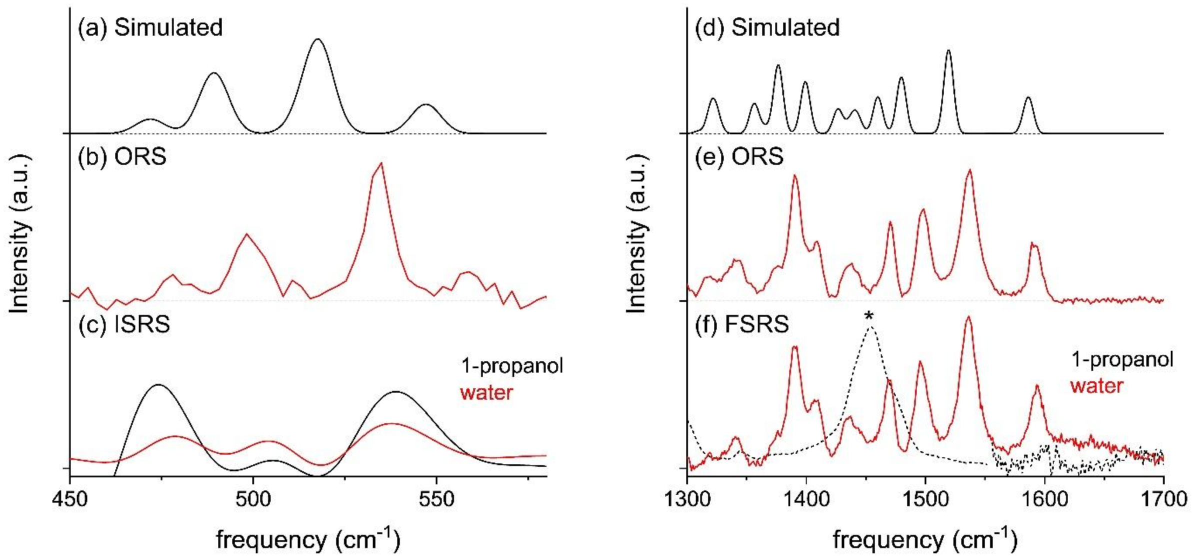

Figure S13. Ground-state Raman spectra of ThT in the frequency ranges of 450−580 cm^{-1} and 1300−1700 cm^{-1}; (a,d) simulated spectrum obtained from the optimized structures by the DFT calculations at B3LYP/6-311G(d,p) with CPCM (1-propanol), (b,e) ordinary Raman spectrum in water (7.5 mM, λ_{exct} = 785 nm), (c,f) ISRS and FSRS spectra in 1-propanol and water.

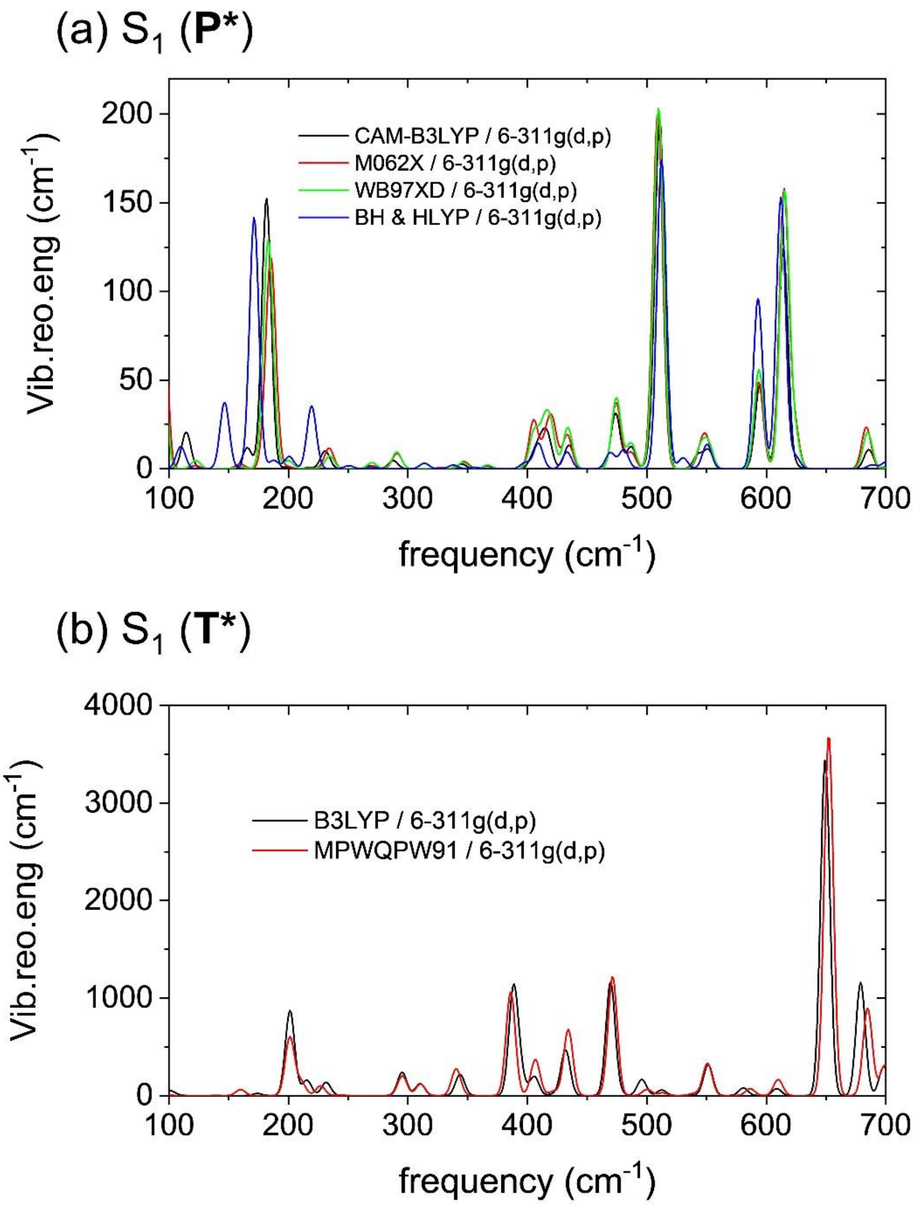

Figure S14. Calculated vibrational reorganization energies between the ground state and partially planar (**P***) and twisted (**T***) geometry in the S_1 state of ThT obtained from TDDFT calculations with the CAM-B3LYP, M062X, WB97XD, BH&HLYP, MPWQPW91, and B3LYP functionals, 6-311G(d,p) basis set, and the CPCM (1-propanol).

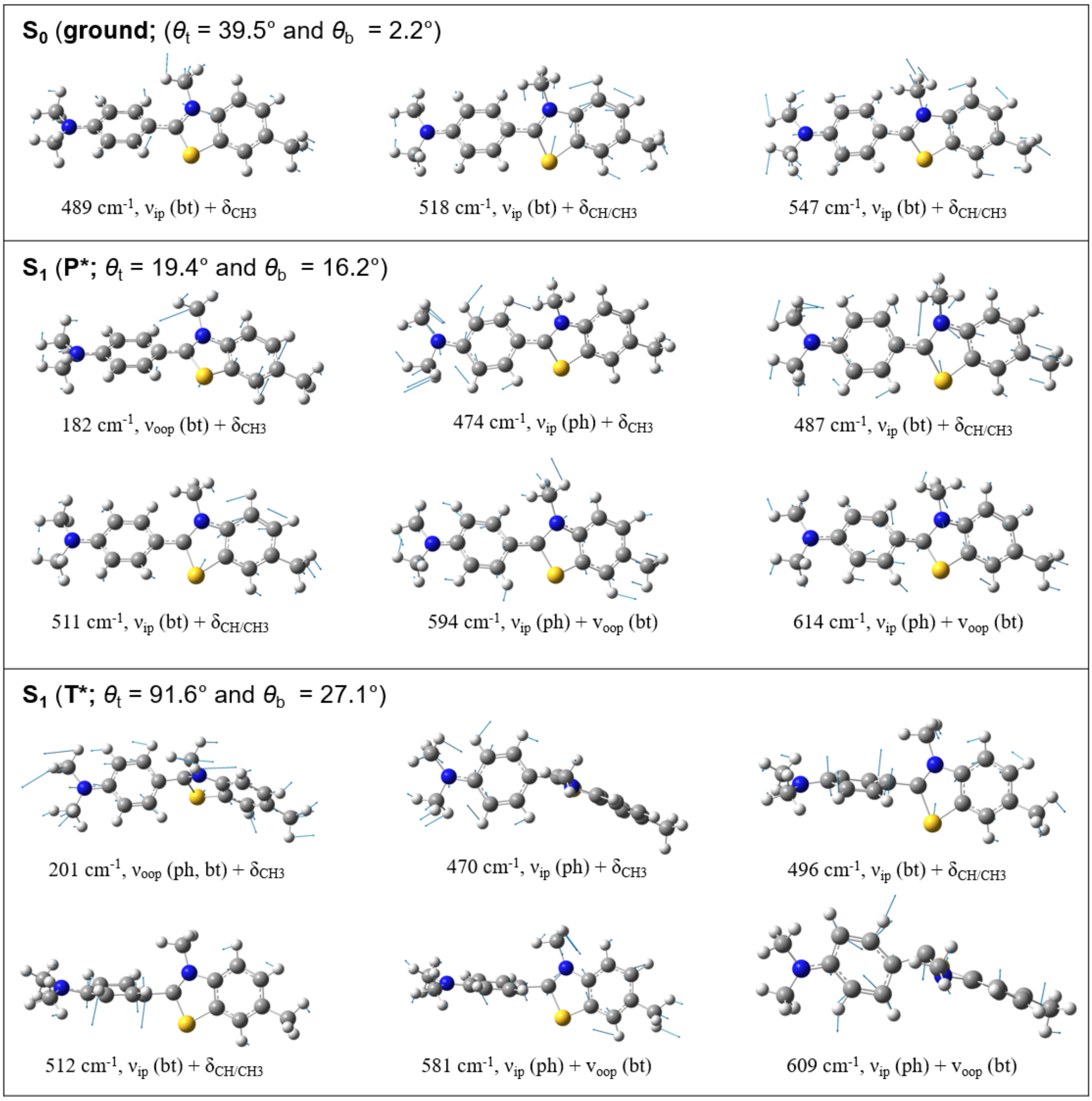

Figure S15. Vibrational normal modes (low frequency region) of ThT in the ground and S_1 state with the partially planar (**P***) and twisted (**T***) geometry obtained from the (TD)DFT calculations with CPCM (1-propanol). The B3LYP/6-311G(d,p) level was used for the ground state and **T*** geometry, and the CAM/B3LYP/6-311G(d,p) level was used for the **P*** geometry. The calculated vibrational frequency of each vibrational mode is displayed and summarized in Tables S3-S4.

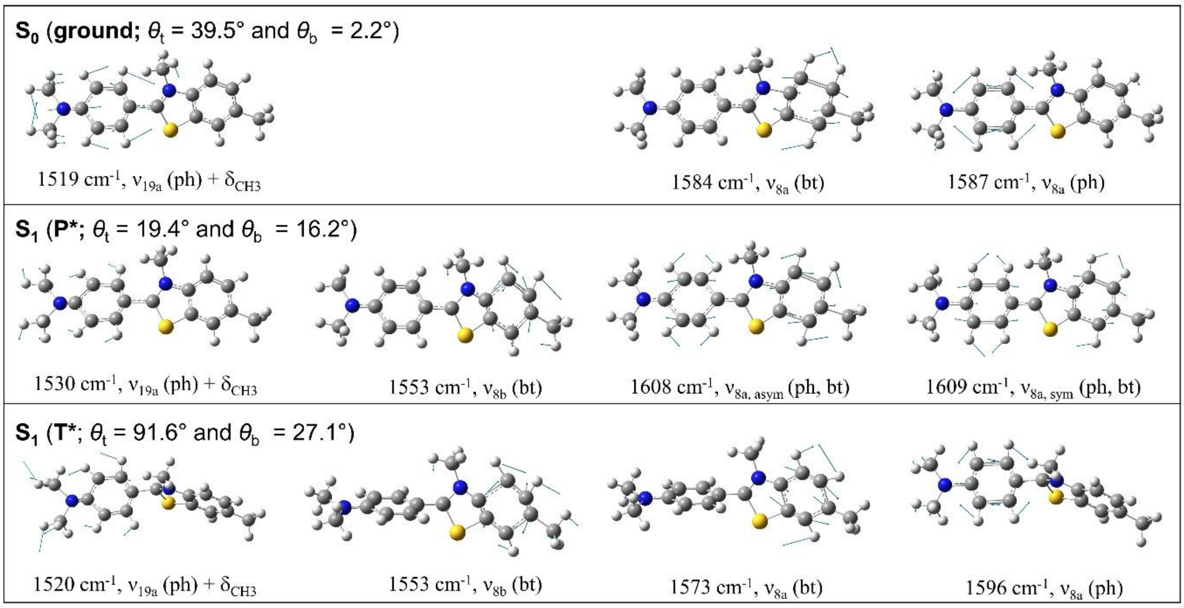

Figure S16. Vibrational normal modes (high frequency region) of ThT in the ground and S_1 state with the partially planar (**P***) and twisted (**T***) geometry obtained from the (TD)DFT calculations with CPCM (1-propanol). The B3LYP/6-311G(d,p) level was used for the ground state and **T*** geometry, and the CAM/B3LYP/6-311G(d,p) level was used for the **P*** geometry. The calculated vibrational frequency of each vibrational mode is displayed and summarized in Tables S3-S4.

Table S2. The optimized structures of ThT in the ground and excited states

optimized geometry	DFT level	bend (θ_b)[1]	twist (θ_t)[2]	energy (eV)[3]
ground state (S_0)	B3LYP / 6-311G(d,p)	2.2	39.5	0
	CAM-B3LYP / 6-311G(d,p)	3.3	37.2	
	M062X / 6-311G(d,p)	3.8	36.5	
	WB87XD / 6-311G(d,p)	3.8	39.2	
	BH&HLYP / 6-311G(d,p)	3.3	36.2	
	MPWQPW91 / 6-311G(d,p)	3.1	34.8	
**P*** (S_1)[4]	CAM-B3LYP / 6-311G(d,p)	16.2	19.4	3.07
	M062X / 6-311G(d,p)	17.4	20.1	3.05
	WB87XD / 6-311G(d,p)	17.1	19.9	3.10
	BH&HLYP / 6-311G(d,p)	15.5	18.8	3.19
**T*** (S_1)[4]	B3LYP / 6-311G(d,p)	27.1	91.6	2.47
	MPWQPW91 / 6-311G(d,p)	27.7	92.5	2.69

[1] The bend (θ_b) angle denotes the angle between the benzothiazole and connected phenyl planes as shown in Figure 1(a).

[2] The twist (θ_t) angle measures the rotation of dimethylaniline group with respect to the benzothiazole group as shown in Figure 1(a).

[3] The ground-state energy (eV) obtained with each DFT level is set as zero. The relative energies of the **P*** and **T*** geometries are given with respect to the ground-state energy obtained with each (TD)DFT level.

[4] **P*** and **T*** geometries denote the partially planar and twisted structures, respectively, obtained from the TDDFT optimization in the S_1 state. The CPCM (1-propanol) was used in all calculations.

Table S3. The vibrational assignments of ThT in the ground state

Experimental[1]			Calculated[2]	Vibrational assignments[3]
1-propanol	methanol	water		
474	478	478	489	ν_{ip} (bt) + δ_{CH3}
505	500	505	518	ν_{ip} (bt) + $\delta_{CH/CH3}$
539	536	537	547	ν_{ip} (bt) + $\delta_{CH/CH3}$
		1341	1321	δ_{CH}
		1372	1356	δ_{CH3}
		1390	1377	$\delta_{CH3/CH}$
		1408	1399	$\delta_{CH3/CH}$
		1435	1426	$\delta_{CH3/CH}$
		1445	1442	δ_{CH3}
		1469	1460	$\delta_{CH3/CH}$
		1496	1480	$\delta_{CH3/CH}$
		1536	1519	ν_{19a} (ph) + δ_{CH3}
		1593	1584	ν_{8a} (bt)
			1587	ν_{8a} (ph)

[1] (*below 700 cm^{-1}*) from the ISRS results in 1-propanol, methanol, and water; (*above 800 cm^{-1}*) from the FSRS results in water.

[2] The optimized structures of thioflavin T in the ground state were obtained from the DFT simulations at the B3LYP/6-311G(d,p) with CPCM (1-propanol), and calculated vibrational frequencies at the B3LYP/6-311G(d,p) level with the scaling factor of 0.967.

[3] ν_{ip} denote in-plane deformation, and ν and δ denote stretching and deformation, respectively. The 'ph' and 'bt' refer to the dimethylaniline group and benzothiazole ring, respectively.

Table S4. The vibrational assignments of ThT in the S_1 excited state

Experimental[1] (1-propanol)		Calculated[2]		Vibrational assignments[3]
Intermediate	S_1-ICT	**P*** geometry	**T*** geometry	
200		182		ν_{oop} (bt) + δ_{CH3}
	191		201	ν_{oop} (ph, bt) + δ_{CH3}
473	475	474	470	ν_{ip} (ph) + δ_{CH3}
505	508	487	496	ν_{ip} (bt) + $\delta_{CH/CH3}$
537	536	511	512	ν_{ip} (bt) + $\delta_{CH/CH3}$
624	616	594/614	581/609	ν_{ip} (ph) + ν_{oop} (bt)
1515	1513	1530	1520	ν_{19a} (ph) + δ_{CH3}
1540		1553	1553	ν_{8b} (bt)
	1570		1596	ν_{8a} (ph)
1573		1608/1609		ν_{8a} (ph, bt)

[1] The experimental frequencies below 1000 cm^{-1} were extracted from the ISRS results of ThT in 1-propanol, and those over 1000 cm^{-1} were extracted from the FSRS results of ThT in 1-propanol.

[2] The optimized structures of ThT in S_1 excited state with the partially planar (**P***) and twisted (**T***) molecular geometry of ThT were obtained from the DFT/TDDFT simulations at the CAM-B3LYP/6-311G(d,p) levels and B3LYP/6-311G(d,p) (planar geometry) with CPCM model (1-propanol), respectively, and calculated vibrational frequencies at the B3LYP/6-311G(d,p) level with a scaling factor of 0.967.

[3] ν_{oop} and ν_{ip} denote out-of-plane and in-plane deformations, respectively. ν and δ denote stretching and bending, respectively. The ph and bt refer to the dimethylaniline and benzothiazole groups, respectively.

6. References

(1) Snellenburg, J. J.; Laptenok, S. P.; Seger, R.; Mullen, K. M.; Stokkun, I. H. M. Glotaran : A Java -Based Graphical User Interface for the R Package TIMP. *J. Stat. Softw.* **2012**, *49,* 1-22.

(2) Amdursky, N.; Erez, Y.; Huppert, D. Molecular Rotors: What Lies Behind the High Sensitivity of the Thioflavin-T Fluorescent Marker. *Acc. Chem. Res.* **2012**, *45* (9), 1548-1557.

(3) Ren, H.; Fingerhut, B. P.; Mukamel, S. Time Resolved Photoelectron Spectroscopy of Thioflavin T Photoisomerization: A Simulation Study. *J. Phys. Chem. A* **2013**, *117* (29), 6096-6104.

(4) Ghosh, R.; Palit, D. K. Ultrafast Twisting Dynamics of Thioflavin-T: Spectroscopy of the Twisted Intramolecular Charge-Transfer State. *ChemPhysChem* **2014**, *15* (18), 4126-4131.

(5) Biancardi, A.; Biver, T.; Mennucci, B. Fluorescent dyes in the context of DNA-binding: The case of Thioflavin T. *Int. J. Quantum Chem.* **2017**, *117* (8), e25349.

(6) Kim, J.; Kim, D. E.; Joo, T. Excited-State Dynamics of Thioflavin T: Planar Stable Intermediate Revealed by Nuclear Wave Packet Spectroscopies. *J. Phys. Chem. A* **2018**, *122* (5), 1283-1290.

(7) Frisch, M. J.; Trucks, G. W.; Schlegel, H. B.; Scuseria, G. E.; Robb, M. A.; Cheeseman, J. R.; Scalmani, G.; Barone, V.; Mennucci, B.; Petersson, G. A.; et al. *Gaussian 09, Revision B.01*; 2009.

(8) Walker, M.; Harvey, A. J. A.; Sen, A.; Dessent, C. E. H. Performance of M06, M06-2X, and M06-HF Density Functionals for Conformationally Flexible Anionic Clusters: M06 Functionals Perform Better than B3LYP for a Model System with Dispersion and Ionic Hydrogen-Bonding Interactions. *J. Phys. Chem. A* **2013**, *117* (47), 12590-12600.

(9) Josa, D.; Otero, J. R.; Cabaleiro Lago, E. M. A DFT study of substituent effects in corannulene dimers. *Phys. Chem. Chem. Phys.* **2011**, *13* (47), 21139-21145.

(10) Becke, A. D. A new mixing of Hartree–Fock and local density-functional theories. *J. Chem. Phys.* **1993**, *98* (2), 1372-1377.

(11) Zhao, Y.; Truhlar, D. G. Hybrid Meta Density Functional Theory Methods for Thermochemistry, Thermochemical Kinetics, and Noncovalent Interactions: The MPW1B95 and MPWB1K Models and Comparative Assessments for Hydrogen Bonding and van der Waals Interactions. *J. Phys. Chem. A* **2004**, *108* (33), 6908-6918.

(12) Irikura, K. K.; Johnson Iii, R. D.; Kacker, R. N. Uncertainties in scaling factors for ab initio vibrational frequencies. *J. Phys. Chem. A* **2005**, *109* (37), 8430-8437.

(13) Merrick, J. P.; Moran, D.; Radom, L. An Evaluation of Harmonic Vibrational Frequency Scale Factors. *J. Phys. Chem. A* **2007**, *111* (45), 11683-11700.

(14) Reimers, J. R. A practical method for the use of curvilinear coordinates in calculations of normal-mode-projected displacements and Duschinsky rotation matrices for large molecules. *J. Chem. Phys.* **2001**, *115* (20), 9103-9109.
